# A mobile restriction–modification system provides phage defence and resolves an epigenetic conflict with an antagonistic endonuclease

**DOI:** 10.1093/nar/gkac147

**Published:** 2022-03-14

**Authors:** Nils Birkholz, Simon A Jackson, Robert D Fagerlund, Peter C Fineran

**Affiliations:** Department of Microbiology and Immunology, University of Otago, PO Box 56, Dunedin 9054, New Zealand; Bioprotection Aotearoa, University of Otago, PO Box 56, Dunedin 9054, New Zealand; Department of Microbiology and Immunology, University of Otago, PO Box 56, Dunedin 9054, New Zealand; Bioprotection Aotearoa, University of Otago, PO Box 56, Dunedin 9054, New Zealand; Genetics Otago, University of Otago, PO Box 56, Dunedin 9054, New Zealand; Department of Microbiology and Immunology, University of Otago, PO Box 56, Dunedin 9054, New Zealand; Bioprotection Aotearoa, University of Otago, PO Box 56, Dunedin 9054, New Zealand; Genetics Otago, University of Otago, PO Box 56, Dunedin 9054, New Zealand; Department of Microbiology and Immunology, University of Otago, PO Box 56, Dunedin 9054, New Zealand; Bioprotection Aotearoa, University of Otago, PO Box 56, Dunedin 9054, New Zealand; Genetics Otago, University of Otago, PO Box 56, Dunedin 9054, New Zealand

## Abstract

Epigenetic DNA methylation plays an important role in bacteria by influencing gene expression and allowing discrimination between self-DNA and intruders such as phages and plasmids. Restriction–modification (RM) systems use a methyltransferase (MTase) to modify a specific sequence motif, thus protecting host DNA from cleavage by a cognate restriction endonuclease (REase) while leaving invading DNA vulnerable. Other REases occur solitarily and cleave methylated DNA. REases and RM systems are frequently mobile, influencing horizontal gene transfer by altering the compatibility of the host for foreign DNA uptake. However, whether mobile defence systems affect pre-existing host defences remains obscure. Here, we reveal an epigenetic conflict between an RM system (PcaRCI) and a methylation-dependent REase (PcaRCII) in the plant pathogen *Pectobacterium carotovorum* RC5297. The PcaRCI RM system provides potent protection against unmethylated plasmids and phages, but its methylation motif is targeted by the methylation-dependent PcaRCII. This potentially lethal co-existence is enabled through epigenetic silencing of the PcaRCII-encoding gene via promoter methylation by the PcaRCI MTase. Comparative genome analyses suggest that the PcaRCII-encoding gene was already present and was silenced upon establishment of the PcaRCI system. These findings provide a striking example for selfishness of RM systems and intracellular competition between different defences.

## Graphical Abstract

**Graphical Abstract:**
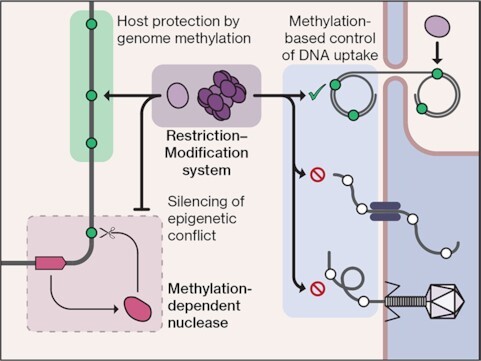
A mobile restriction–modification system provides phage defence and resolves an epigenetic conflict with an antagonistic endonuclease.

## INTRODUCTION

Epigenetic modifications—which are heritable but do not change the base sequence of DNA—govern diverse processes in higher organisms, such as development or the emergence of disease (1–3). However, epigenetics is also of key importance in bacteria (4,5). The most common epigenetic mark is DNA methylation, catalysed by methyltransferases (MTases) and known to occur at adenine residues as N6-methyladenine (m6A) or at cytosine residues as 5-methylcytosine (5mC) or N4-methylcytosine (4mC) (6). Bacterial MTases may act on their own as solitary enzymes, such as the DNA adenine MTase (Dam) and DNA cytosine MTase (Dcm) from *Escherichia coli*, the cell-cycle-regulating MTase (CcrM) from *Caulobacter crescentus* or the *Clostridioides difficile* adenine MTase A (CamA). Solitary MTases can fulfil a variety of roles, for example in DNA replication (7,8), mismatch repair (9,10), cell cycle progression (11), stress response (12,13) or pathogenesis (14).

Many MTases occur in association with restriction endonucleases (REases), forming restriction–modification (RM) systems. Although some of these RM MTases are also known to affect the cellular transcriptome (15–20), a major function of RM systems is widely acknowledged to be protection against invaders such as bacteriophages (phages) or plasmids (21). MTases of RM systems methylate a specific base within a target motif, whereas the REase cleaves DNA after recognition of the same, unmethylated sequence. Therefore, the bacterial genome is protected from cleavage due to methylation, whereas unmethylated intruders are degraded by the REase (22). Four types of RM systems have been defined based on their gene composition, target recognition and cleavage sites (23). Types I, II and III can be considered ‘bona-fide’ RM systems because they consist of MTases and associated REases (24–26), whereas in Type IV, REases occur solitarily and cleave DNA methylated by a noncognate MTase (27). Hence, RM systems and Type IV REases also play a crucial role in controlling horizontal gene transfer (HGT) based on the DNA methylation status of the donor and the REases in the recipient (28–31). Interestingly, RM systems themselves can be subject to HGT (32–35) but might be excluded by REases already present in the recipient cell (36,37). In agreement, the MTase of the StyLTI RM system from *Salmonella enterica* was shown to elicit toxicity when combined with the *E. coli* Type IV REase Mrr, and loci encoding homologs of these proteins appear to be mutually exclusive in the genomes of various strains (38). This suggests that epigenetically incompatible systems cannot stably co-exist in the same host. However, whether this is always the case or whether mechanisms exist to maintain such incompatible, competing systems remains obscure.

Here, we report the discovery and characterization of an antagonistic epigenetic interaction between an RM system and a Type IV REase in *Pectobacterium carotovorum*, an economically important pathogen causing soft rot disease in several crop plants (39). The RM system provides potent protection against plasmids and phages lacking the cognate methylation pattern. In contrast, the Type IV REase degrades DNA carrying the methylation pattern of the RM system. This potentially lethal genomic conflict is resolved through epigenetic repression of the Type IV REase gene by the RM MTase. Comparative analyses of different *P. carotovorum* genomes suggest that the RM system was acquired more recently than the Type IV locus, necessitating silencing of the latter. These findings highlight the selfish character of RM systems and show that different defence systems in the same genome do not necessarily co-exist or complement each other without conflict but may instead be in competition for their own maintenance.

## MATERIALS AND METHODS

### Bacterial strains and growth conditions

Strains used in this study are summarised in Supplementary Table S1, with the construction of strains detailed in the Supplementary Methods. Unless otherwise noted, *Escherichia coli*, *Pectobacterium carotovorum* and *Pectobacterium atrosepticum* strains were grown at 37, 30 and 25°C, respectively, either in lysogeny broth (LB) at 180 rpm or on LB-agar (LBA) plates containing 1.5% (w/v) agar. If applicable, antibiotics and supplements were added at the following concentrations: ampicillin (Ap), 100 μg/ml; chloramphenicol (Cm), 25 μg/ml; kanamycin (Km), 50 μg/ml; δ-aminolevulinic acid (ALA), 50 μg/ml; isopropyl β-d-1-thiogalactopyranoside (IPTG), 50 μM; l-arabinose, 0.05% (w/v). Bacterial growth was measured as the optical density at 600 nm (OD_600_) using a Jenway 6300 Spectrophotometer.

### DNA isolation and manipulation

Oligonucleotides used in this study are listed in Supplementary Table S2. Plasmid DNA was extracted from overnight cultures using the Zyppy Plasmid Miniprep Kit (Zymo Research) and confirmed by DNA sequencing. Plasmids used are listed in Supplementary Table S3, with the construction of new plasmids outlined in the Supplementary Methods. Restriction digests, ligations and *E. coli* transformations were performed using standard techniques. DNA from PCRs and agarose gels was purified using the Illustra GFX PCR DNA and Gel Band Purification Kit (GE Healthcare). Polymerases, restriction enzymes and T4 ligase were obtained from New England Biolabs or Thermo Scientific.

### DNA and protein sequence analyses

DNA sequence analyses were performed using Geneious Prime 11.0.4 software (https://www.geneious.com/). Promoter elements were identified using BPROM (40) and by comparison with established consensus sequences. For comparative genome analyses, genomes of the following *P. carotovorum* strains were retrieved from GenBank (41) in addition to RC5297 (accession number CP045097) and ZM1 (CP045098) (retrieval date 4 October 2021): 2A (CP066552), BP201601.1 (CP034236), JR1.1 (CP034237), PC1 (CP001657), PCC21 (CP003776), PCCS1 (CP063773), WPP14 (CP051652), XP-13 (CP063242). Average Nucleotide Identity (ANI) was calculated using the Kostas Lab ANI matrix calculator with default settings (42). Genome alignments were generated using the Mauve Multiple Genome Alignment tool (43). Protein BLAST (https://blast.ncbi.nlm.nih.gov/), HHPred (44,45) and Phyre2 (46) were used for protein sequence analyses, identification of protein homologs and structure predictions, respectively.

### Preparation of electrocompetent *P. carotovorum* cells and electroporation

For the preparation of electrocompetent *P. carotovorum* cells, an overnight culture of the desired strain was used to inoculate 25 ml LB broth containing the appropriate antibiotics and supplements. The culture was incubated at 25°C with shaking until it reached an OD_600_ of 0.6–0.8. Cells were then pelleted by centrifugation (2220 *g*, 4°C, 10 min) and washed twice in ice-cold water and once in 10% glycerol (v/v). Finally, the pellet was resuspended in 1 ml 10% glycerol (v/v) and 50 μl aliquots of competent cells were stored at −80°C. For transformations, 100 ng plasmid DNA was added to thawed competent cells on ice. After 30 min incubation on ice, electroporation was carried out using a Bio-Rad GenePulser Xcell system (at 1800 V, 25 μF, 200 Ω) in Bio-Rad electroporation cuvettes with a 0.1 cm electrode gap, followed by 2 h recovery in LB at 30°C and 180 rpm. Ten-fold dilutions of the transformed cells were spread on LBA plates with the appropriate antibiotics and supplements.

### Transformation assay

To compare the transformability of plasmids into different backgrounds, purified plasmids were quantified by three concentration measurements of a NanoDrop One spectrophotometer (Thermo Fisher) and, based on the mean value, adjusted to 100 ng/μl. Transformations were carried out by electroporation as described above. The transformed cells were resuspended in 1 ml LB and recovered for 2 h at 30°C. A 10-fold dilution series was prepared and 100 μl of each dilution was spread, or 10 μl of each dilution was spotted, on LBA containing the appropriate antibiotic(s). Transformant numbers were calculated as colony-forming units (CFU) per ml culture per μg plasmid DNA added.

### Conjugation efficiency assay

*Escherichia coli* ST18 carrying the desired plasmid was used as the donor strain for conjugation efficiency assays. Overnight cultures of the donor and recipient strains were washed twice in LB + ALA and the OD_600_ was adjusted to 1. Equal volumes of donor and recipient were mixed and 30 μl spots on LBA + ALA were incubated overnight at 30°C. Spots were resuspended in 1 ml phosphate-buffered saline (PBS) and a 10-fold dilution series was made in PBS. Next, 10 μl of each dilution were spotted on LBA (for total colony counts) and LBA + the appropriate antibiotic (for transconjugant counts). Conjugation efficiency was determined as the ratio of transconjugants to total colonies.

### Bacteriophage isolation and titration

An overnight culture of the phage host strain (*Pca*^wt^, *Pca*^ΔR^ or *Pca*^ΔRM^) was grown and 100 μl were added to 4 ml soft LB-agar (containing 0.35% agar (w/v)), together with 100 μl of 10-fold dilutions of the phage stock to be amplified. The mixture was poured onto an LBA plate and incubated overnight. From a plate with near-confluent lysis, the soft agar layer was scraped using a sterile microscope slide and transferred into a sterile JA20 centrifuge tube. The plate was rinsed with 3 ml phage buffer (10 mM Tris-HCl [pH 7.5], 10 mM MgSO_4_, 0.01% [w/v] gelatine) and the liquid was added to the centrifuge tube. After adding 500 μl chloroform, the tube was vortexed and centrifuged at 2200 *g* for 20 min at 4°C. The supernatant was transferred into a sterile universal and 100 μl chloroform were added to maintain sterility. Phage stocks were stored at 4°C. To determine the phage titre on a given host strain, agar overlays were prepared as described above. Alternatively, 10 μl spots of phage dilutions were placed on agar overlays containing the host strain. The titre was determined as the number of plaque-forming units (PFU) per ml.

### Extraction of genomic DNA

For extraction of genomic DNA (gDNA) for Nanopore genome sequencing, overnight cultures of *Pca*^wt^ and *Pca*^ΔRM^ were grown and gDNA was isolated using the DNeasy Blood & Tissue Kit (Qiagen). Extracted gDNA was further purified using AMPure XP beads (Beckman Coulter) according to the manufacturer’s instructions. The concentration and purity of gDNA was assessed using a NanoDrop Spectrophotometer and the Qubit dsDNA HS Assay Kit (Thermo Fisher). For PacBio genome sequencing, gDNA was extracted from *Pca*^wt^ and ZM1 using cetrimonium bromide (CTAB) (referred to below as the CTAB protocol). A pellet of 3 ml overnight culture of the desired strain was washed twice in 5 M NaCl. The washed pellet was resuspended in 1 ml freshly prepared lysis buffer (50 mM Tris-HCl [pH 8.0], 20 mM NaCl, 2 mM ethylenediaminetetraacetic acid [EDTA], 2% SDS [w/v] and 1 mg/ml protease K in dH_2_O) and 1 ml preheated (65°C) CTAB buffer (100 mM Tris-HCl [pH 8.0], 20 mM EDTA, 1.4 M NaCl, 20 g/L CTAB in dH_2_O) was added. The mixture was incubated for 1 h at 65°C, followed by addition of an equal volume of phenol:chloroform:isoamyl alcohol (25:24:1) and thorough mixing. The sample was centrifuged at 4000 *g* for 5 min at 4°C, and the supernatant was extracted twice with equal volumes of chloroform:isoamyl alcohol (24:1). DNA in the final supernatant was precipitated with 1/10 volume 3 M sodium acetate (pH 5.2) and two volumes ice-cold ethanol and the mixture was incubated at −20°C for 20 min, then pelleted by centrifugation at 10 000 *g* and 4°C for 5 min. The resulting pellet was resuspended in 500 μl Tris-EDTA (TE) buffer and 0.02 mg RNase A was added, followed by incubation at 37°C for 30 min. DNA was extracted multiple times with equal volumes of chloroform:isoamyl alcohol (24:1) until the interface between the phases was clear. The final aqueous phase was precipitated with 1/10 volume of 3 M sodium acetate (pH 5.2) and 2 volumes ice-cold ethanol and incubated at −20°C for 20 min. The sample was centrifuged at 20 000 *g* and 4°C for 10 min and the pellet washed twice with ice-cold 70% ethanol. The final pellet was resuspended in 250 μl TE buffer. DNA concentration was quantified using Qubit.

### Whole-genome sequencing

PacBio RSII sequencing was performed by Macrogen Oceania, South Korea, using gDNA extracted with the CTAB protocol. For Oxford Nanopore sequencing, libraries were prepared from gDNA extracted with the DNeasy Blood & Tissue Kit using the Nanopore Rapid Barcoding Kit according to the manufacturer’s instructions. Nanopore data were obtained using a MinION R9.4.1 flow cell. Base-calling was performed using Guppy (Oxford Nanopore Technologies) and demultiplexing using DeepBinner (47). Tombo was used to detect modified bases by comparison to the reference signal models for unmodified, 6mA or 5mC bases (48).

### RNA extraction and sequencing

For isolation of total RNA from *Pca*^wt^ and *Pca*^ΔRM^, 25 ml of LB were inoculated with 250 μl of overnight cultures of the respective strains and incubated for 6 h at 25°C, up to OD_600_ values between 0.65 and 0.75. Next, 1 ml of each culture was centrifuged for 1 min at 17 000 *g*. The supernatant was discarded and the pellet resuspended in 1 ml RNAlater (Invitrogen) and stored at −20°C. RNA extraction was performed using the RNeasy Mini Kit (Qiagen). Residual gDNA was removed by treatment with TurboDNase (Thermo Fisher) as per the manufacturer’s instructions, and absence of gDNA was confirmed by PCR using primers PF4821+PF4822. RNA purity, integrity and concentration were determined using a NanoDrop One Spectrophotometer (Thermo Fisher) and the Agilent 2100 Bioanalyzer system with an RNA Nano chip. Library preparation using the QIAseq Stranded RNA Library kit (Qiagen), rRNA depletion using the QIAseq FastSelect kit (Qiagen) and RNA sequencing was carried out by the Microbial Genome Sequencing (MiGS) Center (Pittsburgh, USA). Libraries were sequenced at a depth of 14.6–18.0 million reads and 75-bp reads were returned as adapter-trimmed demultiplexed sequences in FASTQ format.

### RNA sequencing analysis

Quality of RNA sequencing was assessed by running FastQC on the returned FASTQ files (https://www.bioinformatics.babraham.ac.uk/projects/fastqc/). Raw reads were aligned to the *P. carotovorum* RC5297 genome using Bowtie 2 with default parameters (49) and the alignment was converted to BAM format using SAMtools (50). Up- and downregulated transcripts were identified in RStudio using DESeq2 (51), with a false discovery rate of 5%. An output list was generated containing the following parameters for each gene: the base mean as a measure of read abundance, the log_2_-fold change with its associated standard error, and a *P* value adjusted for multiple testing (*P*_adj_).

### Reporter assay

Reporter assays were performed as in (52) and involved plasmids for arabinose-inducible expression of *pcaIM* and/or reporter plasmids with *eyfp* under the control of the *pcaIIR* promoter. To determine promoter activity and the effect of promoter mutations in different backgrounds, plasmids pPF1439 (no promoter), pPF2860 (wild-type promoter) or pPF2861 (point-mutated promoter) were transformed into *Pca*^wt^ or *Pca*^ΔRM^. For complementation experiments, these strains additionally contained the *pcaIM* expression plasmid pPF2865 or the corresponding empty-vector control (pBAD30). Overnight cultures of the strains to be tested were grown in 96-well plates in an IncuMix incubator shaker (Select BioProduct) at 1200 rpm at 30°C. After adjusting the OD_600_ to 0.05 in fresh media containing the appropriate antibiotics as well as IPTG (one-plasmid assay) or IPTG and arabinose (two-plasmid assay), the cultures were incubated for 20 h and fluorescence of plasmid-encoded mCherry and eYFP was measured by flow cytometry in a BD LSRFortessa Cell Analyzer. First, cells were gated based on forward and side scatter area. A 610/20-nm bandpass filter with a detector gain of 606 V was used to detect mCherry-positive cells, which were then analysed for eYFP levels with a 530/30-nm bandpass filter and detector gain of 600 V. Median eYFP fluorescence intensity was measured for six biological replicates; measurements outside of three standard deviations around the mean were omitted as outliers.

### Competition assay

Competition assays were based on previous studies (53,54). The strains *Pca*^wt^ and *Pca*^ΔRM^ were transformed with plasmids encoding mCherry (pPF1739) or ZsGreen (pPF1751), with fluorophore expression inducible by IPTG. Overnight cultures of the strains to be competed against each other were grown with Km (for plasmid maintenance) and the OD_600_ was adjusted to 1. An equal mix of both strains was used to inoculate (at 1:100 dilution) 5 ml fresh LB with Km. The culture was grown at 30°C and passaged for 3 days by inoculating (at 1:100 dilution) fresh LB with Km. At the beginning of the experiment and at the end of each passaging cycle, a dilution series of the mixed culture was plated on LBA with Km and IPTG. The fraction of *Pca*^wt^ cells was determined once fluorophore expression was readily discernible on the plates, after approximately 48 h incubation at 30°C. Relative fitness (*F*) of *Pca*^wt^ was determined using the equation *F* = *N*_t_ × (1 – *N*_0_)/[*N*_0_ × (1 – *N*_t_)], where *N*_0_ and *N*_t_ represent the fraction of *Pca*^wt^ at the beginning of the experiment and after the time *t* (1, 2 or 3 days), respectively.

## RESULTS

### *Pectobacterium carotovorum* RC5297 discriminates self and foreign DNA

**Figure 1.**
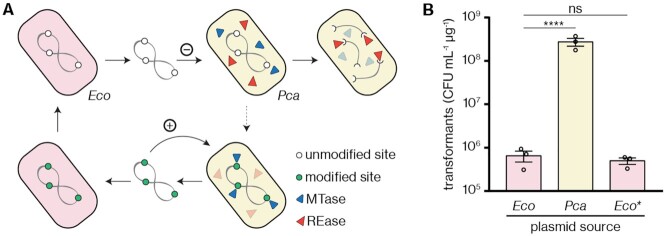
*Pectobacterium carotovorum* RC5297 exhibits a restriction–modification phenotype. (**A**) Workflow to test for RM system activity. The strain in question (*Pca*) is transformed with a plasmid isolated from an unrelated strain, *E. coli* DH5α (*Eco*). If an RM system is active, plasmids are expected to be taken up with low efficiency (**–**), with the dashed arrow illustrating the rare event that plasmids become modified prior to restriction. However, plasmids isolated from *Pca* itself can be transformed more efficiently (**+**) due to the presence of compatible DNA modifications. Passaging of the plasmid through *E. coli* reverts this phenotype due to modification loss. (**B**) *Pca* transformant counts in colony-forming units (CFU) per mL per μg of a plasmid (pTRB30) isolated from *E. coli* or *Pca*, or from *E. coli* after passaging (Eco*). Results are the mean and associated standard error, with independent replicates represented by dots. Statistical significance was assessed using Dunnett's multiple comparisons test on log_10_-transformed data. ns: *P* > 0.05, ****P < 0.0001.

We previously used *Pectobacterium carotovorum* RC5297 (hereafter *Pca*) as a permissive host to study anti-CRISPR regulation by the anti-CRISPR-associated protein Aca2 from phage ZF40 (52). However, we initially observed that plasmid uptake by *Pca* was substantially lower than by the related *Pectobacterium atrosepticum* SCRI1043 (Supplementary Figure S1). We hypothesised that *Pca* encodes a defence system as a barrier against plasmid uptake. To determine whether the defence system acts via an epigenetic mechanism, such as for RM systems, we performed a series of classical transformation experiments (Figure 1A) (55). This strategy assumes that if an epigenetic mechanism is present, plasmids isolated from *Pca* will carry an epigenetic mark—for example, the methylation pattern of the RM MTase. Therefore, these modified plasmids should be taken up by *Pca* with greater efficiency than plasmids isolated from a strain lacking the epigenetic modification (Figure 1A). Indeed, plasmids isolated from *Pca* transformants exhibited much higher re-transformation rates than the same plasmids isolated from *E. coli* DH5α, suggesting that plasmid modification takes place in *Pca* (Figure 1B). To rule out the possibility that a mutation rather than an epigenetic modification was responsible for this, we passaged plasmids isolated from *Pca* through *E. coli*, which led to a subsequent decrease in transformation efficiency into *Pca* (Figure 1B). These findings show that *Pca* can discriminate self and foreign DNA, likely through RM system activity.

### A restriction–modification system inhibits plasmid acquisition and phage infection

**Figure 2.**
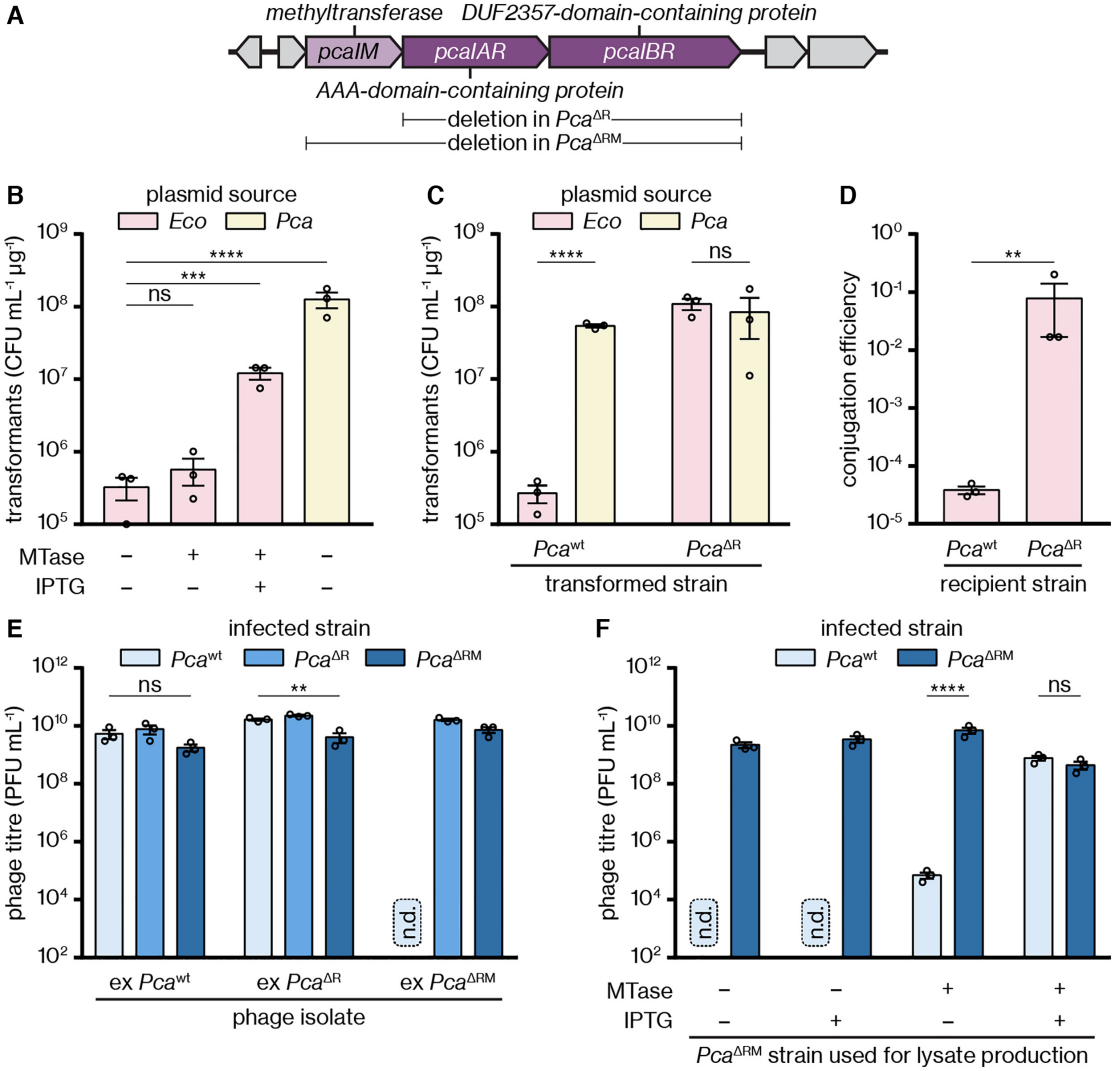
A restriction–modification system in *Pca* inhibits plasmid uptake and phage infection. (**A**) Locus encoding the RM system (shades of purple); refer to Supplementary Figure S5B for more details on neighbouring genes. Genes deleted in *Pca*^ΔR^ and *Pca*^ΔRM^ are indicated below. (**B**) *Pca* transformant counts when transformed with an empty vector (–MTase, pTRB30) or an MTase expression plasmid (+MTase, pPF1375) replicated in *E. coli* in the presence or absence of IPTG induction, compared to an uninduced empty vector isolated from *Pca*. (**C**) *Pca*^wt^ or *Pca*^ΔR^ transformant counts when transformed with a plasmid (pTRB30) isolated from *E. coli* or *Pca*^wt^. (**D**) Conjugation efficiency of a plasmid (pPF953) into the *Pca*^wt^ or *Pca*^ΔR^ backgrounds; *E. coli* ST18 was used as the donor strain. (**E**) Titres of phage ZF40 isolated from the *Pca*^wt^, *Pca*^ΔR^ or *Pca*^ΔRM^ backgrounds when infecting the same three strains. (**F**) Titres of phage ZF40 lysates prepared in *Pca*^ΔRM^ in the presence of an empty vector (pTRB30) or an MTase expression plasmid (pPF1375), and in the presence or absence of IPTG induction, when infecting *Pca*^wt^ or *Pca*^ΔR^. n.d., not detected, below limit of detection (10^2^ PFU mL^–1^). Panels (B)–(F) display the mean and associated standard error, with independent replicates represented by dots. Statistical significance was assessed using Dunnett’s multiple comparisons test (B), two-tailed unpaired *t*-tests (C,D,F), or one-way ANOVA (E) on log_10_-transformed data. ns: *P* > 0.05, **P < 0.01, ***P < 0.001, ****P < 0.0001.

To identify the system responsible for the RM phenotype, we performed PacBio whole-genome sequencing of *Pca* and searched for genes encoding likely plasmid defence systems. This revealed that *Pca* encodes a putative RM system composed of an MTase gene and two genes encoding a bipartite REase (Figure 2A). One REase subunit contained a putative AAA+ GTPase domain, while the other subunit was annotated as DUF2357, which is known to be an endonuclease domain (56). This gene combination is present in other REases (57). To test whether this RM system was responsible for the observed RM phenotype, we first cloned the MTase gene into an expression plasmid and induced expression in *E. coli*. The presumably self-methylated plasmid could be transformed into *Pca* more efficiently than the same plasmid grown without induction or an empty vector control (Figure 2B), indicating methylation-dependent protection from degradation. To test whether exclusion of foreign DNA was caused by the predicted bipartite REase, we performed a knockout of the two putative REase genes, resulting in a *Pca*^ΔR^ strain. This strain displayed high transformant counts regardless of the plasmid source (*E. coli* or *Pca*) (Figure 2C). Likewise, efficiency of conjugation from the commonly used donor strain *E. coli* ST18 (58,59) to the *Pca*^ΔR^ recipient was strongly enhanced compared with the wild-type recipient (Figure 2D), confirming a role of the REase in defence.

We next wanted to find out whether the RM system also protects *Pca* against infection by *P. carotovorum* phage ZF40. We generated a *Pca*^ΔRM^ strain, which enabled preparation of ZF40 phages lacking the MTase modification. In contrast, phages isolated from *Pca*^wt^ or *Pca*^ΔR^ strains would be methylated. Phages isolated from *Pca*^wt^ or *Pca*^ΔR^ displayed high infectivity on any host strain (*Pca*^wt^, *Pca*^ΔR^ or *Pca*^ΔRM^) whereas phages isolated from *Pca*^ΔRM^ failed to infect *Pca*^wt^—the only strain of the three capable of producing the REase of the RM system (Figure 2E). However, infectivity on *Pca*^wt^ was restored when the MTase was provided *in trans* during production of the phage lysate (Figure 2F). Taken together, our results show that this RM system provides *Pca* with strong protection against plasmid uptake by transformation and conjugation as well as against phage infection. In accordance with the proposed nomenclature for RM systems (23), we name the MTase M.PcaRCI (encoded by the gene *pcaRCIM*), and the components of the REase R.PcaRCIA (*pcaRCIAR*) and R.PcaRCIB (*pcaRCIBR*), or for short PcaRCIA and PcaRCIB, respectively (Figure 2A). For conciseness, throughout the rest of this paper we will omit the strain designation ‘RC’ (for RC5297) and refer to the system and its components simply as PcaI/*pcaI*, but note that this name is officially listed in the RM system database REBASE to specify a system from *Pelobacter carbinolicus* (60,61).

### The RM system components are homologs of the Dcm MTase and the McrBC REase

**Figure 3.**
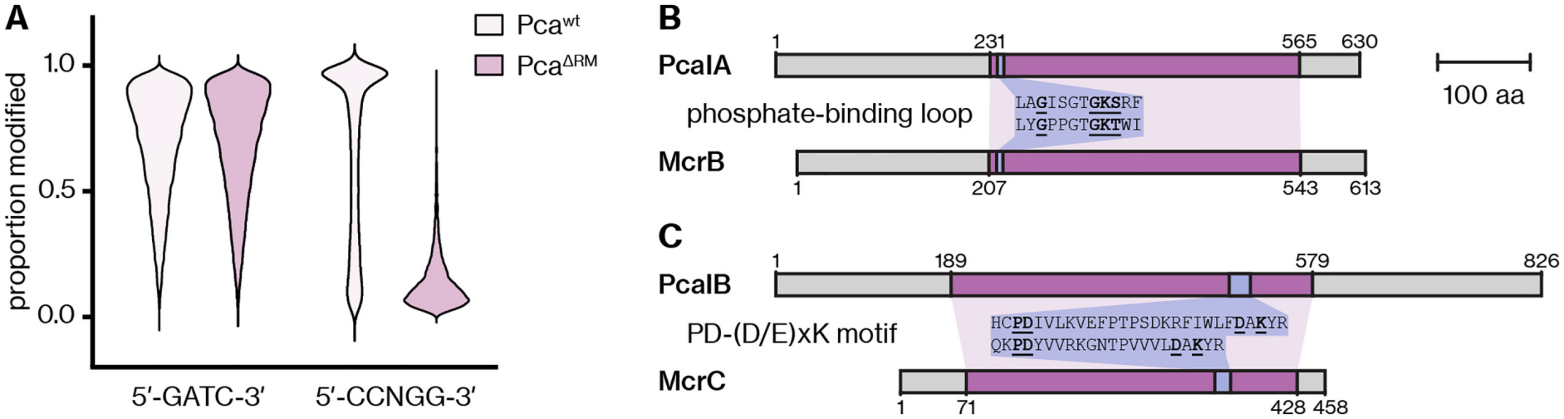
The PcaI RM system consists of a MTase methylating 5′-CCNGG-3′ motifs and a REase resembling McrBC. (**A**) The proportion of 5′-GATC-3′ and 5′-CCNGG-3′ motifs identified by Nanopore sequencing as being methylated in *Pca*^wt^ and *Pca*^ΔRM^. (**B**) Domain alignment of PcaIA and *T. gammatolerans* McrB. Sequences aligned with high confidence using Phyre2 are shown in purple, with the phosphate-binding loop highlighted in blue and a sequence alignment shown in the centre. (**C**) Domain alignment of PcaIB and *T. gammatolerans* McrC. The PD-(D/E)xK motif is highlighted in blue, with a sequence alignment shown in the centre. For (B) and (C), a scale bar is given to indicate a length of 100 amino acids.

We next aimed to characterize the components and recognition site of the RM system. Our initial PacBio sequencing not only provided the genome sequence of *Pca*^wt^ but also revealed 6mA methylation at 5′-GATC-3′ sites, which we attributed to a Dam homolog encoded in the *Pca* genome (locus tag F9W95_01820). Because PacBio sequencing has low sensitivity for 5mC methylation (18,62,63), we performed Oxford Nanopore MinION sequencing of the *Pca*^wt^ and *Pca*^ΔRM^ genomes. In addition to the 5′-GATC-3′ modification in both strains, we detected 5mC methylation in 5′-CCNGG-3′ contexts in *Pca*^wt^ but not *Pca*^ΔRM^ (Figure 3A). Therefore, the 5′-CCNGG-3′ motif is the recognition site of M.PcaI. This site is similar to the 5′-CCWGG-3′ motif (where W is A or T) recognized by *E. coli* Dcm, which shares 38% amino-acid identity with M.PcaI (Supplementary Figure S2) but does not have any other close homologs in the *Pca* genome. Note that in our previous transformation and conjugation experiments (see Figures 1B, 2B–D), plasmids from *dcm*^+^ strains (*E. coli* DH5α or ST18), which are methylated at 5′-CCWGG-3′ sites, could still be targeted, implying that protection only at these sites is insufficient. Restriction was likely elicited by unmethylated 5′-CCSGG-3′ sites (where S is C or G), several of which are present within each of the plasmids tested (see Supplementary Figure S1).

The REase component of the RM system likely consists of the proteins PcaIA and PcaIB (Figure 2A). PcaIA is predicted to contain an AAA+ domain for binding and hydrolysis of ATP or GTP. A Phyre2 search (46) revealed with high confidence (99.9%) that a distinct domain of several McrB homologs, such as from *Thermococcus gammatolerans* or *E. coli*, displays similarity to part of the predicted PcaIA structure. McrB is the GTPase component of the composite REase McrBC, which recognizes methylated 5′-RC-3′ sites (where R is A or G) (64) and is therefore a Type IV REase not normally associated with a cognate MTase. McrB contains an N-terminal DNA-binding domain and a C-terminal GTPase domain with a phosphate-binding loop (65). The similarity to PcaIA was restricted to the C-terminus, suggesting divergence in the DNA-binding domain (Figure 3B). Using the same structural prediction and homology search approach as above, PcaIB was found to share homology with McrC, the endonuclease component of McrBC (99.4% confidence). However, similarity was restricted to the endonuclease domain including a PD-(D/E)xK motif, which is involved in nucleic acid cleavage (56) (Figure 3C). PcaIB possesses large N- and C-terminal extensions of unknown function, which are absent in McrC. In conclusion, the PcaIAB REase shares regions of homology with McrBC but has an unrelated DNA-binding domain, which likely accounts for the polar differences in target specificity (unmethylated instead of methylated DNA).

### Genome methylation by M.PcaI inhibits an alternative defence mechanism

**Figure 4.**
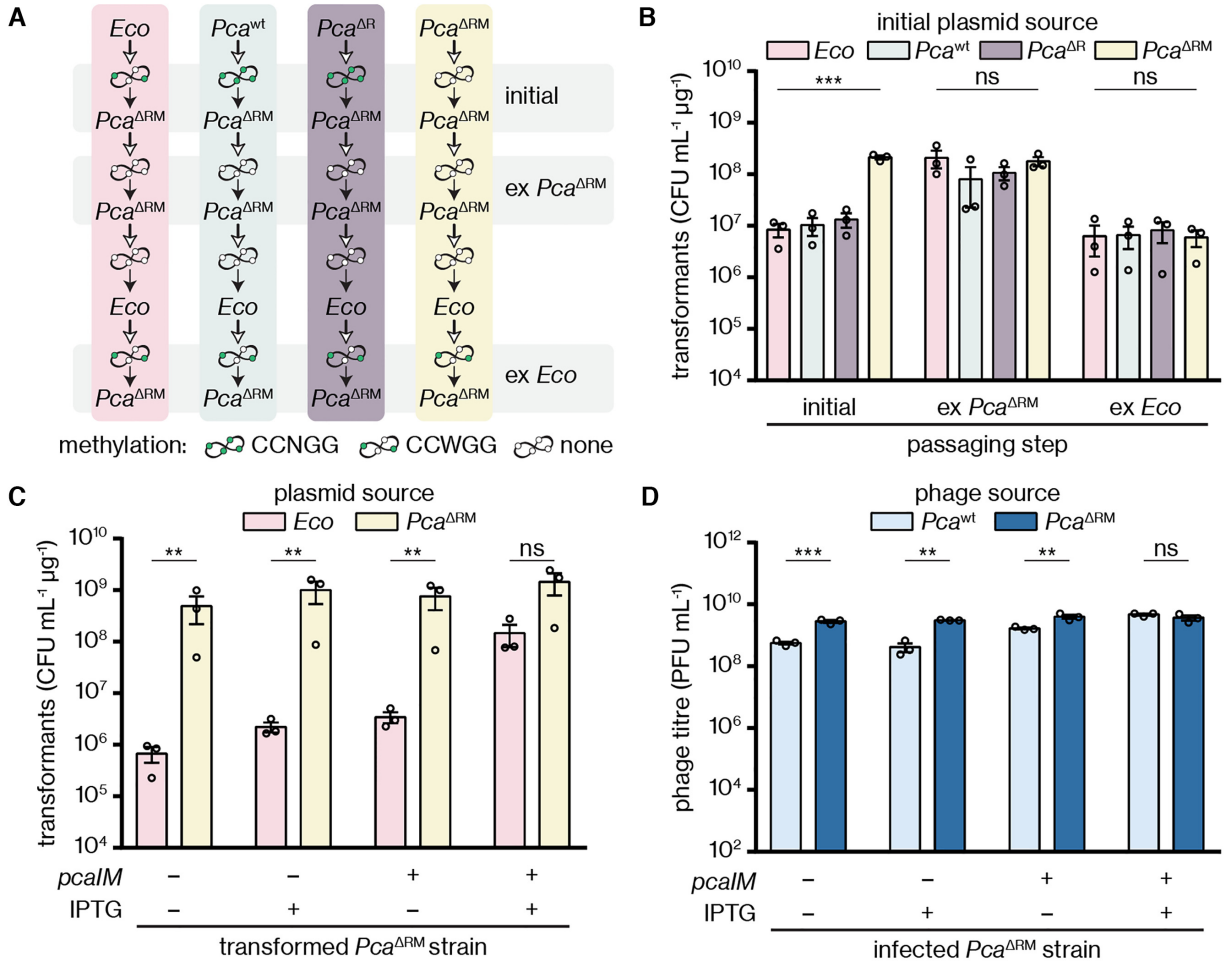
The MTase M.PcaI suppresses a secondary defence phenotype. (**A**) Schematic illustrating the passaging experiment in (B). Plasmid isolation is indicated with an empty arrow and transformation with a solid arrow. Expected methylation states are illustrated with green (methylated, by M.PcaI or *E. coli* Dcm) or white (unmethylated) dots as indicated below; the number of methylation sites is indicative only. First, plasmids (pTRB30) were isolated from four different hosts (colour-coded to match the bars in (B)), followed by transformation into *Pca*^ΔRM^ (initial). Plasmids were isolated from all *Pca*^ΔRM^ strains and re-transformed into *Pca*^ΔRM^ (ex *Pca*^ΔRM^). Plasmids were isolated from all *Pca*^ΔRM^ strains, passaged through *E. coli*, and re-transformed into *Pca*^ΔRM^ (ex *Eco*). (**B**) *Pca*^ΔRM^ transformant counts throughout the passaging experiment illustrated in (A). (**C**) Transformant counts upon transformation of plasmids (pBAD30) from different sources into *Pca*^ΔRM^ hosts already containing an IPTG-inducible *pcaIM* expression (or empty) vector (pPF1375 and pTRB30, respectively), with the competent cells prepared in the presence or absence of IPTG induction. (**D**) Titres of ZF40 phage stocks from *Pca*^wt^ or *Pca*^ΔRM^ upon infection of *Pca*^ΔRM^ hosts with or without *pcaIM* complementation by pPF1375 or the empty-vector control pTRB30. Panels (B)–(D) display the mean and associated standard error, with independent replicates represented by dots. Statistical significance was assessed using one-way ANOVA (B) or two-tailed unpaired *t*-tests (C,D) on log_10_-transformed data; ns: *P* > 0.05, **P ≤ 0.01, ***P ≤ 0.001.

In the process of confirming that plasmids isolated from *Pca*^ΔRM^ lose their protection against the PcaI RM system, we observed that the plasmid source still affected the resulting number of *Pca*^ΔRM^ transformants: plasmids isolated from *Pca*^ΔRM^ itself yielded higher transformant counts than plasmids isolated from other strains (Figure 4A,B). This was surprising, since in the absence of the RM system we expected similar outcomes regardless of the plasmid source. Moreover, when we isolated plasmids from the resulting *Pca*^ΔRM^ transformants and then re-transformed *Pca*^ΔRM^, we observed high transformant counts in all cases. However, this effect was lost after subsequent passaging through *E. coli* (Figure 4A,B). We reasoned that this return to low transformation efficiencies might be due to re-acquisition of a modification that had been lost in *Pca*^ΔRM^, which could be caused, for example, by the *E. coli* MTase Dcm.

Aside from the RM knockout, *Pca*^wt^ and *Pca*^ΔRM^ are isogenic, as confirmed by the genome sequences of both strains. Therefore, we hypothesized that the phenotype observed in *Pca*^ΔRM^ was caused by the absence of the RM system due to a regulatory role of M.PcaI in suppressing a secondary defence system. To test this, we performed MTase complementation in *Pca*^ΔRM^ via IPTG-inducible expression of *pcaIM*. As predicted, this complementation increased the efficiency of transformation with plasmids sourced from *E. coli* by 100-fold (Figure 4C). In contrast, transformation using *Pca*^ΔRM^-sourced plasmids was efficient irrespective of MTase complementation in the *Pca*^ΔRM^ recipient. A similar complementation effect was observed in phage infection assays, where ZF40 sourced from *Pca*^wt^ could infect *Pca*^ΔRM^ at approximately 10-fold higher titres if the MTase was expressed, whereas there was no dependence on MTase expression for ZF40 sourced from *Pca*^ΔRM^ (Figure 4D). Overall, these data suggested the presence of a cryptic epigenetic-based defence system that is active in *Pca*^ΔRM^ but not *Pca*^wt^ or *Pca*^ΔR^.

### A methylation-dependent HNH endonuclease provides defence in *Pca*^ΔRM^

**Figure 5.**
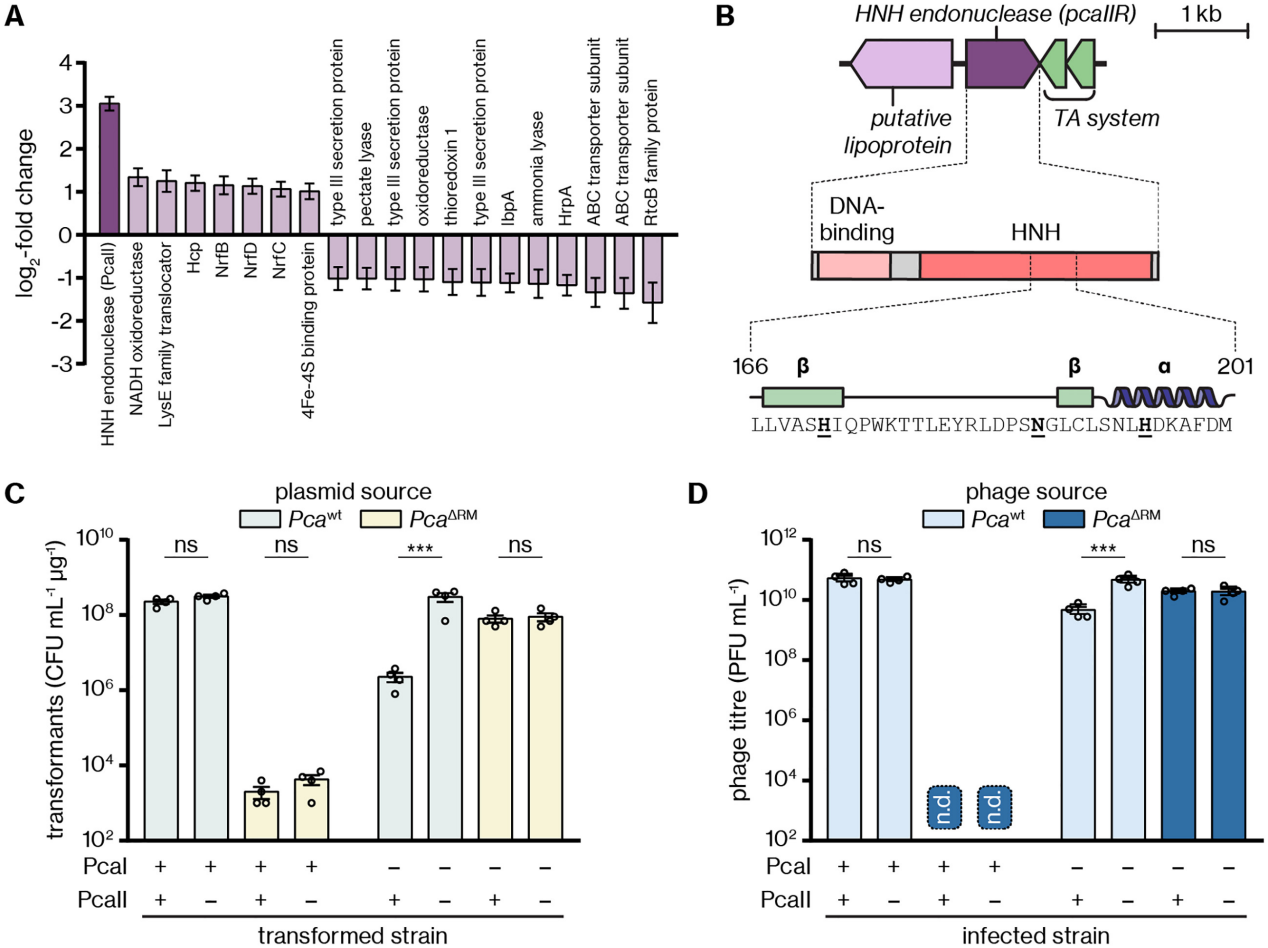
An HNH endonuclease is upregulated and provides secondary defence in *Pca*^ΔRM^. (**A**) Genes significantly up- and downregulated in *Pca*^ΔRM^ compared to *Pca*^wt^, based on five independent replicates per strain, with the respective protein products indicated. Only genes/proteins with a log_2_-fold change >1 (and the associated standard error) are listed; refer to Supplementary Tables S4 and S5 for more details. (**B**) The locus surrounding the upregulated HNH endonuclease gene (*pcaIIR*), with the domain architecture and HNH domain of the protein (catalytic residues in bold) highlighted underneath. (**C**) Transformant counts upon transformation of plasmids (pTRB30) from *Pca*^wt^ or *Pca*^ΔRM^ into *Pca* hosts with the PcaI RM system and/or the gene encoding the PcaII REase present or knocked out. (**D**) Titres of ZF40 phage stocks from *Pca*^wt^ or *Pca*^ΔRM^ after infection of the same *Pca* strains as in (C). n.d., not detected, below limit of detection (10^2^ PFU mL^–1^). Panels (C) and (D) display the mean and associated standard error, with independent replicates represented by dots. Statistical significance was assessed using two-tailed unpaired *t*-tests on log_10_-transformed data. ns: *P* > 0.05, ***P < 0.001.

Since activity of the cryptic defence mechanism was observable only in the *Pca*^ΔRM^ strain and was abolished by *pcaIM* complementation, it appeared that M.PcaI negatively regulates the expression of this mechanism. To test this, we used RNAseq to analyse the transcriptomes of *Pca*^wt^ and *Pca*^ΔRM^ cultures during exponential growth (equivalent to the competent cells in which the alternative defence phenotype had been observed). Our analysis revealed that 40 genes were significantly (*P*_adj_ < 0.05) upregulated in *Pca*^ΔRM^ compared to *Pca*^wt^ (Supplementary Table S4) and 67 genes were significantly downregulated (Supplementary Table S5). Expression changes for most of these genes were moderate; however, one gene, encoding a putative HNH endonuclease, was strongly upregulated by 8.3-fold in *Pca*^ΔRM^ (log_2_-fold change of 3.05) (Figure 5A). We termed this gene and the encoded protein *pcaRCIIR* and R.PcaRCII (or PcaRCII for short), respectively; as with the PcaI RM system, we will omit the official strain designation ‘RC’ for the remainder of this paper. No MTase gene was found in the vicinity of *pcaIIR* (Figure 5B), suggesting that the encoded enzyme is not part of an RM system. Phyre2 and HHpred searches showed resemblance of the C-terminal half of PcaII to HNH domains of methylation-dependent HNH endonucleases such as VcaM4I (66) and TagI (67) (Supplementary Figure S3) but also to HNH domains of methylation-independent enzymes such as the CRISPR-associated nuclease Cas9. Consistent with other HNH nucleases (68), the HNH motif of PcaII is embedded in a predicted ββα topology (Figure 5B). The N-terminus of the protein resembles the three-helical bundle of the human telomeric protein hTRF1 (69) (Supplementary Figure S3) and is therefore a predicted DNA-binding domain.

To test whether PcaII was responsible for the defence phenotype observed in *Pca*^ΔRM^, we generated *pcaIIR* knockouts in the *Pca*^wt^ and *Pca*^ΔRM^ backgrounds and examined the resulting strains in transformation (Figure 5C) and phage infection assays (Figure 5D). In both assays, the *pcaIIR* knockout did not significantly affect the function of the PcaI RM system. However, with the PcaI RM system deleted, plasmids or phages methylated at 5′-CCNGG-3′ sites (sourced from *Pca*^wt^) were restricted in the presence, but not in the absence, of *pcaIIR* (Figure 5C,D). These results demonstrate that the HNH endonuclease PcaII can provide secondary defence in *Pca*^ΔRM^ by targeting methylated plasmids and phages and confirm that this defence is repressed in the presence of the PcaI RM system.

### Methylation by M.PcaI represses the *pcaIIR* promoter

**Figure 6.**
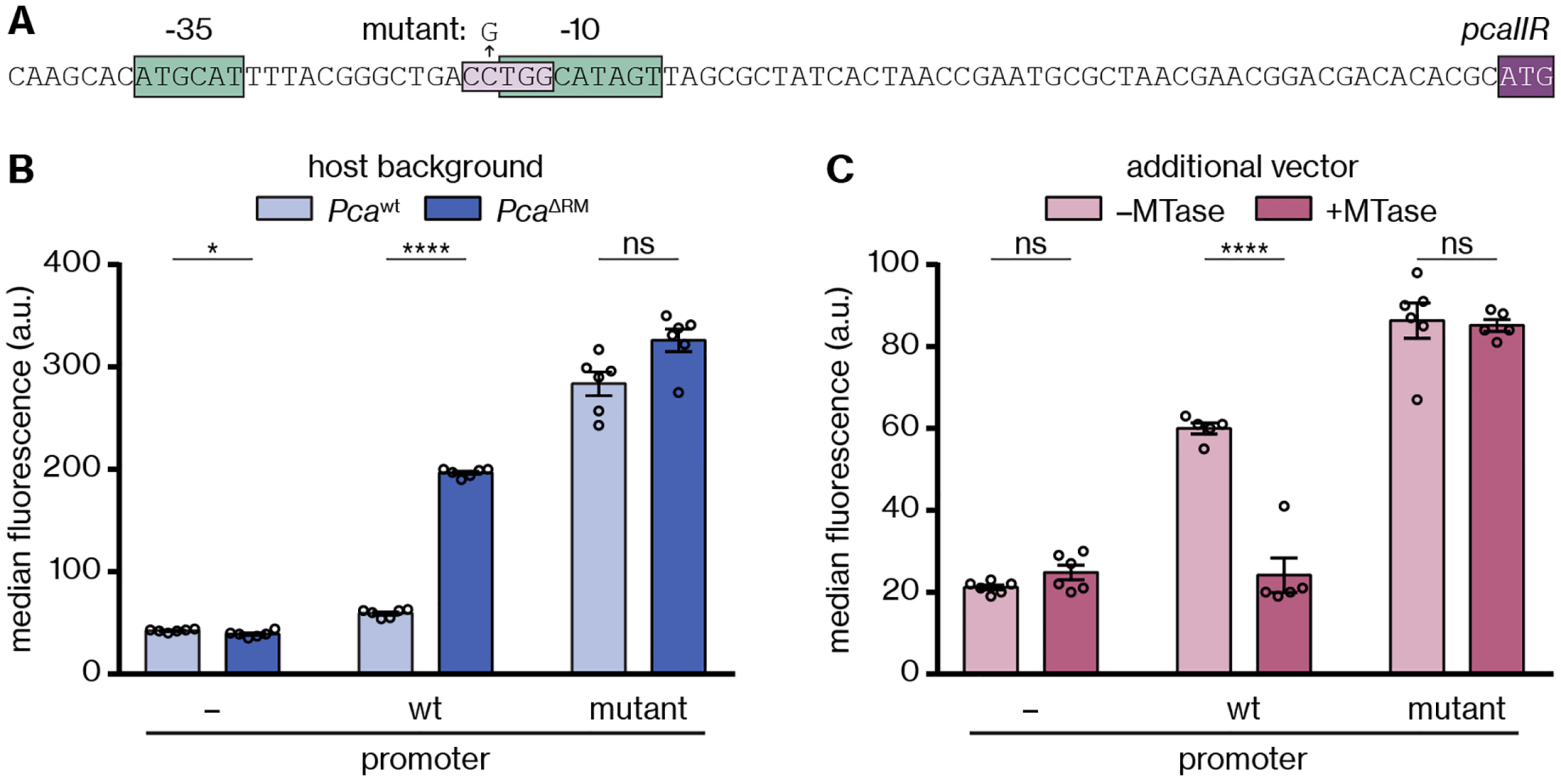
M.PcaI-mediated methylation within the *pcaIIR* promoter leads to repression of gene expression. (**A**) Overview of the *pcaIIR* promoter, with predicted -35 and (extended) -10 regions highlighted in green. The M.PcaI methylation site is shown in light purple, the *pcaIIR* start codon in dark purple; the point mutation investigated in the reporter assays is indicated with an arrow. (**B**) Activity of the wild-type (wt) *pcaIIR* promoter (pPF2860) or a promoter variant with a 5′-CGTGG-3′ point mutation (mutant) in the methylation motif (pPF2861), compared to an empty vector control (pPF1439), in the *Pca*^wt^ or *Pca*^ΔRM^ background, determined as the median eYFP fluorescence. (**C**) Activity of the same promoter variants in the *Pca*^ΔRM^ background in the presence of an additional plasmid for expression of *pcaIM* or an empty vector (pPF2865 or pBAD30, respectively). Panels (B) and (C) display the mean and associated standard error, with independent replicates represented by dots. Statistical significance was assessed using two-tailed unpaired *t*-tests; ns: *P* > 0.05, *P ≤ 0.05, ****P ≤ 0.0001.

Our previous results suggested that repression of *pcaIIR* in *Pca*^wt^ is mediated through methylation by M.PcaI. To clarify whether repression occurred directly or indirectly, we analysed the promoter region of *pcaIIR* and identified a potential methylation motif (5′-CCTGG-3′) overlapping with the predicted extended -10 element (Figure 6A). To test the activity of the *pcaIIR* promoter, we fused the 100 bp preceding the start codon to *eyfp* on a reporter plasmid. We observed robust eYFP fluorescence in *Pca*^ΔRM^, but only background-level fluorescence in *Pca*^wt^ (Figure 6B), consistent with repression of *pcaIIR* in the presence of the RM system. Strikingly, a promoter variant with a point mutation in the 5′-CCTGG-3′ motif—replacing it with 5′-CGTGG-3′—resulted in strong *eyfp* expression in both strains (Figure 6B). Therefore, a single intact methylation site in the *pcaIIR* promoter is essential for repression of gene expression. To confirm that repression is mediated by M.PcaI, we performed the reporter assay with an additional plasmid for *pcaIM* overexpression. In *Pca*^wt^, *pcaIM* overexpression had no effect (Supplementary Figure S4), whereas in *Pca*^ΔRM^, *pcaIM* overexpression restored repression of the wild-type but not the mutated promoter (Figure 6C). These findings demonstrate that methylation of this 5′-CCTGG-3′ motif in the *pcaIIR* promoter by M.PcaI leads to repression of *pcaIIR* expression, which explains the lack of detectable PcaII activity in the presence of the PcaI RM system.

### The RM system is sparse in *P. carotovorum* strains and is part of a variable genomic region

**Figure 7.**
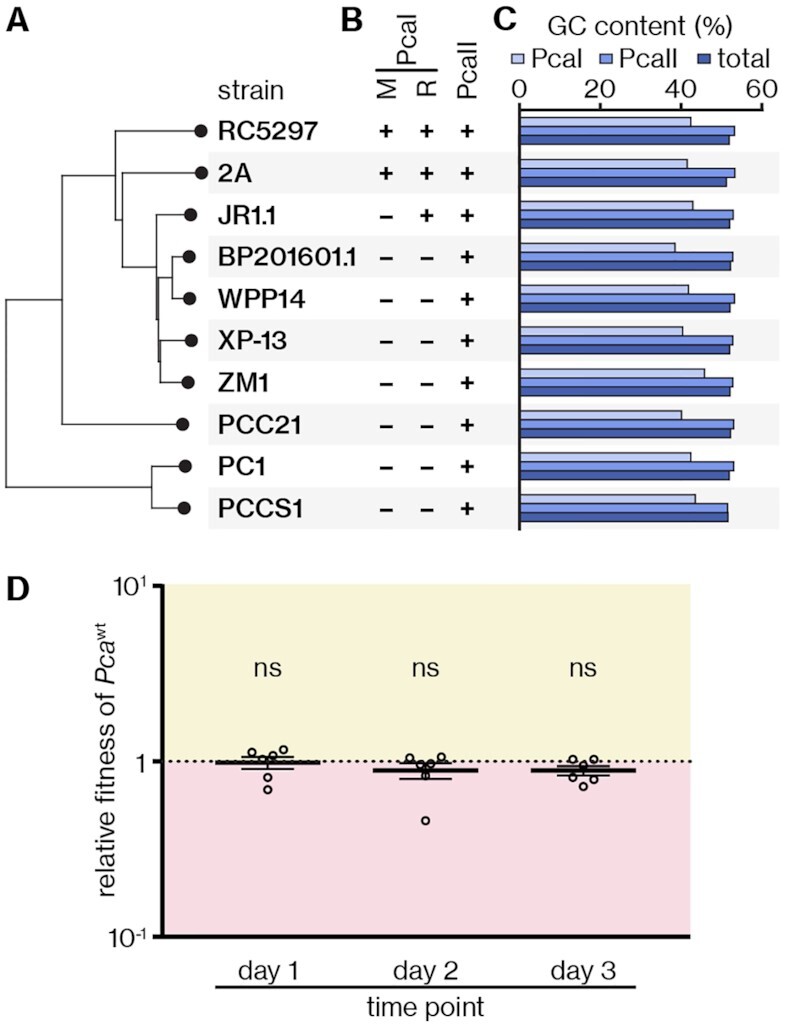
Co-existence of the mobile PcaI RM system and PcaII is infrequent among *P. carotovorum* strains and does not have a detectable fitness cost. (**A**) Distance clustering plot of ten *P. carotovorum* strains based on their pairwise average nucleotide identity (ANI), computed using the BIONJ clustering method ((42), see Supplementary Figure S5A); *E. coli* K12 was used for rooting but was omitted from the figure for clarity. The length of the horizontal lines illustrates evolutionary distance. (**B**) Presence or absence of genes encoding M.PcaI (M) and PcaIAB (R) or PcaII across the different *P. carotovorum* strains. (**C**) GC contents around the PcaI- and PcaII-encoding loci or their equivalents (as displayed in Supplementary Figures S5B,C) as well as whole genomes of the different *P. carotovorum* strains. (**D**) Relative fitness of *Pca*^wt^ (carrying the *zsgreen* expression plasmid pPF1751) when competed against *Pca*^ΔRM^ (carrying the *mcherry* expression plasmid pPF1739) for 3 days (see Supplementary Figure S6 for the same experiment with reciprocal fluorophores). Shown are the mean and associated standard error, with independent replicates represented by dots. Statistical significance was assessed using a one-sample *t*-test against the value 1, indicating no change in relative fitness; ns: *P* > 0.05.

Our results showed that *P. carotovorum* RC5297 encodes two REases with identical or overlapping target sequences but opposing specificities for unmethylated (PcaIAB) or methylated (PcaII) DNA. While the MTase M.PcaI seems to resolve this apparent paradox through methylation and epigenetic repression of the *pcaIIR* promoter, we wondered how this state may have originated. For an unbiased overview of the distribution of loci encoding the PcaI RM system and PcaII, we analysed all *P. carotovorum* genomes listed as ‘complete’ in GenBank (see Materials and Methods) and also performed whole-genome sequencing of *P. carotovorum* ZM1, a lysogen for phage ZF40. Based on average nucleotide identity (ANI) calculations (42) (Supplementary Figure S5A), the resulting set of ten genomes (including *Pca* RC5297) displayed varying degrees of relatedness, with some strains clustering together and others, such as PC1 and PCCS1, appearing more divergent from the rest (Figure 7A). An alignment of the region that, in RC5297, contains the PcaI RM system revealed high variability in terms of composition and size (from 2.0 kb in PCCS1 to 19.6 kb in ZM1), including between closely related strains (99% ANI) such as BP201601.1 (14.2 kb) and WPP14 (5.4 kb) (Supplementary Figure S5B). The complete PcaI RM system was present in only two of the ten strains, RC5297 and 2A (Figure 7B). Despite the variability, a common theme was the presence of genes typical for mobile genetic elements, such as TA system components, MTases and integrases (Supplementary Figure S5B). The locus surrounding *pcaIIR* was much more conserved (Supplementary Figure S5C), with the *pcaIIR* gene present in all strains (Figure 7B). Furthermore, the GC content in the region around the PcaI RM system, or in the equivalent regions in other strains, was generally lower than around the *pcaIIR* locus (39.0–45.9% and 51.5–53.3%, respectively; see Supplementary Figure S5B) or throughout the entire genome (51.1–52.2%) (Figure 7C). Together, these observations suggest that the PcaI RM system is part of a mobile accessory region, whereas *pcaIIR* might be part of the *P. carotovorum* core genome. Therefore, we propose that *pcaIIR* was originally present in RC5297 and was silenced upon acquisition of the PcaI RM system.

### Co-existence of *pcaI* and *pcaII* loci does not have a significant fitness cost

Our phylogenetic analysis suggested that the ancestral strain had *pcaIIR* and, in few cases such as RC5297, later acquired the PcaI RM system. We were interested to see if there was any remaining conflict between these two systems. For example, it is possible that *pcaIIR* repression by M.PcaI in RC5297 is incomplete and occasional production of PcaII leads to genome damage, resulting in decreased fitness compared to a PcaI-less strain. To investigate this, we performed a competition experiment between *Pca*^wt^ and *Pca*^ΔRM^, with the latter representing the state found in the majority of *P. carotovorum* strains due to the absence of the PcaI system. To distinguish between the two strains, we transformed each with a plasmid encoding either mCherry or ZsGreen to allow differentiation after plating on media that supports fluorophore induction. A mixed population of *Pca*^wt^ and *Pca*^ΔRM^ was passaged for 3 days and the fraction of *Pca*^wt^ enumerated daily. Throughout this period, we did not detect a deviation in relative fitness of *Pca*^wt^ from the value 1, indicating the absence of a significant fitness disadvantage compared to the PcaI-less strain under these conditions (Figure 7D and Supplementary Figure S6). Therefore, we propose that the PcaI RM system and *pcaIIR* can co-exist without conflict, most likely enabled through *pcaIIR* promoter repression by M.PcaI.

## DISCUSSION

In this study, our search for a genetic barrier against foreign DNA uptake in *P. carotovorum* RC5297 led to the discovery of the PcaI RM system (officially PcaRCI). We found that this system provides highly potent defence against invasion by plasmid DNA and phages. A REase knockout resulted in a strain that is easily amenable to genetic manipulation and has already served as a model organism to investigate the regulation of the anti-CRISPR gene *acrIF8* (52). Importantly, we showed that the MTase of this system, M.PcaI, epigenetically silences the methylation-dependent REase PcaII (officially PcaRCII), which otherwise targets DNA bearing the M.PcaI methylation mark. Thus, we revealed a striking case of two defence systems in conflict, as well as a mechanism by which such a conflict can be resolved.

**Figure 8.**
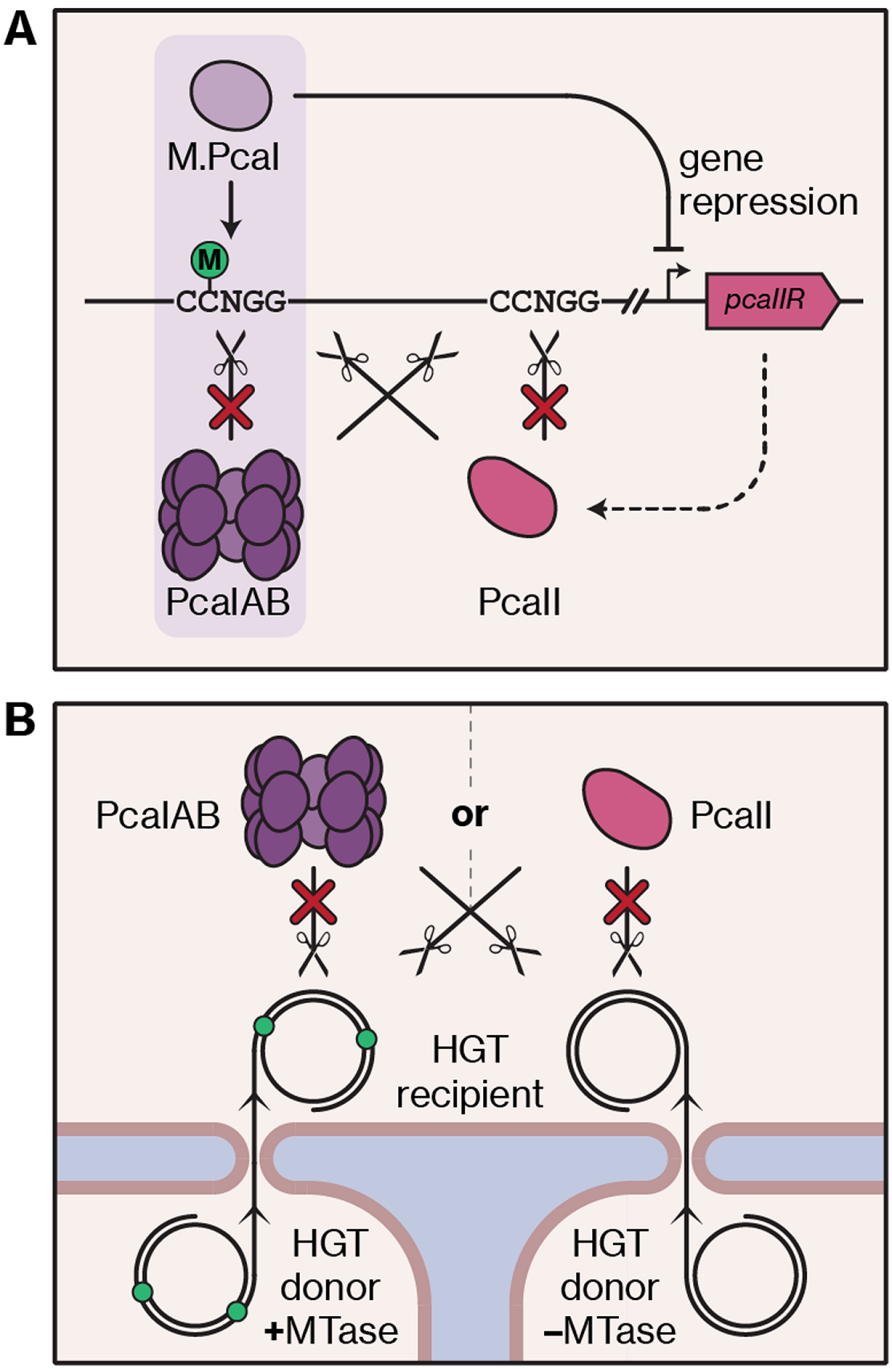
The conflict between the PcaI RM system and PcaII and its potential impact on horizontal gene transfer. (**A**) In *P. carotovorum* RC5297, the REases PcaIAB and PcaII have opposing specificities (unmethylated and methylated DNA, respectively), which necessitates *pcaIIR* silencing by the MTase M.PcaI. The light purple box highlights the components of the PcaI RM system. The dashed arrow represents expression of *pcaIIR* in the absence of methylation. (**B**) Depending on which type of REase is present and/or active in a given strain, this impacts which other strains can serve as donors for horizontal gene transfer (HGT). For example, the presence of PcaIAB restricts uptake of unmethylated DNA but would permit DNA transfer from a strain in which methylation at the correct motif (green dots) takes place. DNA from such a donor strain would, however, be restricted in the presence of PcaII. The tetradecameric architecture of the PcaIAB complex is inferred based on the McrBC architecture proposed by (74,75).

MTases of some RM systems have previously been shown to affect the expression of various genes in their host chromosomes (16–20). The effect of the PcaI RM system on the overall transcriptome was moderate but much more pronounced with regards to the *pcaIIR* gene, whose repression could be pinpointed to methylation at a single site in the promoter. In the absence of repression, the HNH REase PcaII restricts phages and plasmids with 5mC modifications in 5′-CCWGG-3′ and potentially all 5′-CCNGG-3′ contexts, even though its minimal target requirements might be more relaxed; for example, the REase TagI, whose HNH domain is predicted to exhibit structural similarity to that of PcaII, cleaves DNA that is 5mC-methylated by MTases with various distinct target specificities (67). The co-existence of the PcaI RM system and *pcaIIR* in the same genome constitutes a paradoxical scenario because M.PcaI-mediated methylation protects from cleavage by PcaIAB but simultaneously generates targets for PcaII (Figure 8A). Therefore, we propose that repression of *pcaIIR* is absolutely required for cell survival. This would underline the selfish character attributed to RM systems (33,70–73): loss of an RM system will result in post-segregational killing, as remaining MTases cannot keep all recognition sites in a replicating chromosome methylated, leading to cleavage by remaining REases. The co-existence with *pcaIIR* could be interpreted as an additional mechanism to accelerate host killing: gradual de-methylation after loss of the RM locus would allow production of PcaII, which could then target remaining methylated sites. Interestingly, the Type IV REase McrBC was suggested to act as a safeguard against parasitic RM systems because it would kill the host once the new MTase starts methylating the genome (36,37). Given the likelihood that the PcaI RM system was mobile and acquired by a genome already encoding PcaII, it is possible that the latter had a similar function in preventing parasitism but was silenced by M.PcaI before execution.

Clues hinting at mobility of the PcaI RM system might also be found in its composition. Based on the similarity of M.PcaI to *E. coli* Dcm, we expect this enzyme to act as a monomer. In contrast, PcaIAB displays similarity to McrBC, whose subunits assemble into a large tetradecameric complex (74,75). Two such complexes must bind targets up to 3 kb apart, translocate, and collide for DNA cleavage to occur (76). Due to the homology between McrBC and PcaIAB in the domains involved in translocation and cleavage, we speculate that PcaIAB might exhibit a similarly intricate mechanism, despite the stark difference in target specificity (64). Such complexity could aid establishment of the RM system in a new host, as it would skew the race between modification and restriction sharply in favour of the MTase. Interestingly, the LlaJI RM system, containing another McrBC-like REase, is found on a conjugative plasmid (57) and may support the idea that this enzyme architecture lends itself to mobility.

Mobility of the PcaI RM system—or the region in which it is embedded—would be in line with previous findings that RM systems are subject to HGT (32–35). Once established, the RM system would have manifested its addictive character, which would have also drastically changed the types of foreign DNA the cell was able to receive. This cell would no longer be able to take up DNA with unmodified 5′-CCNGG-3′ sites, but because PcaII would not be active anymore, transfer of 5mC-modified DNA would now be possible (Figure 8B). Hence, acquisition of the RM system would isolate the lineage of the recipient cell from RM-negative cells within the population and from other cells with incompatible methylation patterns. The flexibility observed across *P. carotovorum* strains with regards to the presence of the PcaI RM system, and potentially other epigenetic marks, might generate incompatibility groups that allow or prevent HGT between one another. This isolating character was previously suggested as an important function of RM systems (28–31) and could contribute to bacterial sympatric speciation (77,78). Bacterial populations in which subsets with different REase specificities exist might also be more flexible facing phage invasion (79). For example, phages may occasionally escape an RM system if their genomes become modified before REase cleavage. Uncontrolled propagation of such escape phages might be prevented if a subset of the population produced a REase that can target methylated DNA. The co-existence of two systems—mutually exclusive at the cellular but beneficial at the population level—would represent a stark contrast to previously described cases of different defence systems within the same cell complementing each other (80–84).

In conclusion, this study underlines the character of RM systems as mobile addiction modules and reveals a striking example of competition between two defence mechanisms. In an important plant pathogen such as *P. carotovorum* (39), this comes with additional implications. Virulence determinants frequently travel between strains through HGT (85), which will be affected by the defence repertoires and epigenetic compatibility of donor and recipient. Moreover, phages are being used as biocontrol agents to contain bacterial infections (86,87), which might be hindered by the spread of bacterial defence systems. Therefore, our investigation of the interaction between the PcaI RM system and the PcaII REase will contribute to the improvement of such applications.

## DATA AVAILABILITY

The genomes of *P. carotovorum* strains RC5297 and ZM1 are available at GenBank under accession numbers CP045097 and CP045098, respectively. Nanopore sequencing data are available at NCBI’s Sequence Read Archive (88) as part of BioProject PRJNA576076. RNA sequencing data have been deposited in NCBI’s Gene Expression Omnibus (89) and are accessible through GEO Series accession number GSE190077.

## Supplementary Material

gkac147_Supplemental_File
