# Supplementary material for: A mobile restriction–modification system provides phage defence and resolves an epigenetic conflict with an antagonistic endonuclease
Source: Nucleic Acids Res. 2022 Mar 14;50(6):3348–61. doi: 10.1093/nar/gkac147 (PMC8989522; doi:10.1093/nar/gkac147)
Supplement: gkac147_Supplemental_File [file gkac147_supplemental_file.pdf]

SUPPLEMENTARY INFORMATION

A mobile restriction–modification system provides phage defence and resolves an epigenetic conflict with an antagonistic endonuclease

Nils Birkholz[1,2], Simon A Jackson[1,2,3], Robert D Fagerlund[1,2,3], Peter C Fineran[1,2,3,*]

[1] Department of Microbiology and Immunology, University of Otago, Dunedin, New Zealand

[2] Bioprotection Aotearoa, University of Otago, Dunedin, New Zealand

[3] Genetics Otago, University of Otago, Dunedin, New Zealand

* To whom correspondence should be addressed: peter.fineran@otago.ac.nz

Supplementary Table S1: Strains used in this study.

name	genotype/phenotype	reference
Pectobacterium carotovorum		
RC5297	carotovoricin-resistant *P. carotovorum* strain; used as wild type in this study (*Pca*^{wt})	(1)
PCF425	RC5297 derivative with *pcaIAR–pcaIBR* knockout (*Pca*$^{\Delta R}$)	this study
PCF433	RC5297 derivative with *pcaIM–pcaIAR–pcaIBR* restriction–modification system knockout (*Pca*$^{\Delta RM}$)	this study
PCF890	RC5297 derivative with *pcaIIR* knockout	this study
PCF891	PCF433 derivative with *pcaIIR* knockout	
ZM1	lysogen for phage ZM1	(1)
Pectobacterium atrosepticum		
SCRI1043	wild-type *P. atrosepticum* strain	(2)
Escherichia coli		
DH5α	cloning strain; *dam*$^{+}$ *dcm*$^{+}$	(3)
ST18	donor strain for conjugation; λ*pir*$^{+}$; Δ*hemA*; *dam*$^{+}$ *dcm*$^{+}$	(4,5)
**Bacteriophages**		
ZF40	temperate phage, family *Myoviridae*; infects *P. carotovorum* strains	(1)

Supplementary Table S2: Oligonucleotides used in this study.

name	sequence (5′-3′)	functions and relevant restriction sites
PF138	CACACTTTGCTATGCCATAG	fwd screening primer for the pBAD30-derived *pcaIM* expression plasmid pPF2865
PF139	GCTACTGCCGCCAGG	rev screening primer for the pBAD30-derived *pcaIM* expression plasmid pPF2865
PF192	TGAGCGGATAACAATTTCAC	fwd screening primer for the pTRB30-derived *pcaIM* expression plasmid pPF1375
PF209	TCGTCTTCACCTCGAGAAATC	fwd primer for amplification from gBlocks PF5625 and PF5626
PF210	GTCATTACTGGATCTATCAACAGG	rev screening primer for the pTRB30-derived *pcaIM* expression plasmid pPF1375; rev primer for amplification from gBlocks PF5625 and PF5626
PF2241	CCAGCTCGACCAGGATGG	rev screening/sequencing primer for *pcaIIR* promoter reporter plasmids
PF2517	TCCAAGCTTGACTCCTGTTGATAGAT	fwd screening/sequencing primer for *pcaIIR* promoter reporter plasmids
PF2630	TTTCAATTG*AGGAGG*ATGAAAAATAGTTCACAAATTATCAATA	fwd for amplification of *pcaIM* and attachment of *ribosome binding site*; MfeI
PF2631	TTTCTGCAGTTAAAAGTAAGATATTAACTTTCTAGAAATAGCTTC	rev for amplification of *pcaIM*; PstI
PF2698	TTTTGCTAGCATGGAAAGACGGTGAGCTAG	fwd for amplification of upstream flank for *pcaIAR–pcaIBR* knockout; NheI
PF2699	AGGATCCTATCCTCGAGACGAGCATGAGAAGTCCTTTTAAAAGTAAG	rev for amplification of upstream flank for *pcaIAR–pcaIBR* knockout; BamHI, XhoI
PF2700	GCTCGTCTCGAGGATAGGATCCTAATATCACATGCAGGAGAGGGTGA	fwd for amplification of downstream flank for *pcaIAR–pcaIBR* knockout; XhoI, BamHI
PF2701	AAAGATGCATCCAGCTCTGCTACACTGTG	rev for amplification of downstream flank for *pcaIAR–pcaIBR* knockout; NsiI
PF2857	GTCAAAGGCTTAATAAGCC	fwd screening/sequencing primer for *pcaIAR–pcaIBR* knockout
PF2858	GTAGTTCAATACGCCACTG	rev screening/sequencing primer for *pcaIAR–pcaIBR* and *pcaI* RM system knockouts
PF2916	TTTTGCTAGCGTGCAGAATACAATACTTG	fwd for amplification of upstream flank for *pcaI* RM system knockout; NheI
PF2917	TTAGGATCCTATCCTCGAGACGAGCCATTGTTTATAACTACCACG	rev for amplification of upstream flank for *pcaI* RM system knockout; BamHI, XhoI
PF2918	GTAGCGAAGCGATTAGATC	fwd screening/sequencing primer for *pcaI* RM system knockout
PF4821	TTTTGCTAGCCATAAGGTTGCATGAACAAC	fwd for confirming absence of gDNA from extracted RNA
PF4822	TTAGGATCCTATCCTCGAGACGCATTGTGGCATGTGTGAAAC	rev for confirming absence of gDNA from extracted RNA
PF5606	GCCATGCGTGTGTCGTCCGTTCGTTAGCGCATTCGGTTAGTG	rev for amplification of upstream flank for *pcaIIR* knockout
PF5607	GAACGGACGACACACGCATGGCGTAAATACGCTATTGGTAGGTCG	fwd for amplification of downstream flank for *pcaIIR* knockout
PF5616	CACGACGGCTAAACACCTC	fwd screening/sequencing primer for *pcaIIR* knockout
PF5625	TCGTCTTCACCTCGAGAAATCACTAGTCAATTATCCAGCCGCAGGCAAGCACATGCATTTTACGGGCTGACCTGGCATAGTTAGCGCTATCACTAACCGAATGCGCTAACGAACGGACGACACACGCATGGGATCCCCTGTTGATAGATCCAGTAATGAC	gBlock containing wild-type *pcaIIR* promoter; binding sites for PF209&PF210; SpeI, BamHI
PF5626	TCGTCTTCACCTCGAGAAATCACTAGTCAATTATCCAGCCGCAGGCAAGCACATGCATTTTACGGGCTGACGTGGCATAGTTAGCGCTATCACTAACCGAATGCGCTAACGAACGGACGACACACGCATGGGATCCCCTGTTGATAGATCCAGTAATGAC	gBlock containing point-mutated *pcaIIR* promoter; binding sites for PF209&PF210; SpeI, BamHI
PF5627	TTTGAATTC*AGGAGG*ACAGGGATGAAAAATAGTTCACAAATTATCAATA	fwd for amplification of *pcaIM* and attachment of *ribosome binding site*; EcoRI
PF5628	TTTGTCGACTTAAAAGTAAGATATTAACTTTCTAGAAATAGCTTC	rev for amplification of *pcaIM*; SalI
PF5689	TTTTGTCGACGATAGCCCTTCGCGTTAG	fwd for amplification of upstream flank for *pcaIIR* knockout; SalI
PF5690	TTTTCTCGAGGACAGCCCGTATATTGCC	rev for amplification of downstream flank for *pcaIIR* knockout; XhoI
PF5691	GCATATTACCGAATACGCGACAG	rev screening/sequencing primer for *pcaIIR* knockout

Supplementary Table S3: Plasmids used in this study.

name	characteristics	reference
pBAD30	bacterial expression vector, p15A/M13 ori, MCS under P_{araBAD} promoter control, Ap^R	(6)
pQE80L	QIAexpress pQE vectors, ColE1 ori, MCS under T5-lac promoter control, Ap^R	Qiagen
pQE80L-*oriT*-*mcherry*	pQE80L-derivative, *mcherry*, *oriT*, Ap^R; used to test tractability of *Pca*	(7)
pTRB30	pQE80L-derivative, Ap^R replaced with Km^R; used for transformation experiments	(8)
pPF781	pBAD30-derivative, Ap^R replaced with Cm^R, *oriT*; used to test tractability of *Pca*	(9)
pPF953	pQE80L-*oriT-mcherry* derivative, *oriT*, Ap^R; used to test tractability of *Pca*	(10)
pPF1117	suicide vector for allelic exchange mutagenesis; ori6RK, *oriT*, Cm^R	(10)
pPF1121	ColE1 ori, *oriT*, Km^R; used to test tractability of *Pca*	Simon Jackson, unpublished
pPF1122	ColE1 ori, *oriT*, Cm^R; used to test tractability of *Pca*	Simon Jackson, unpublished
pPF1375	pTRB30-derived IPTG-inducible *pcaIM* expression plasmid	this study
pPF1424	pPF1117-derived, containing knockout cassette for *pcaIAR–pcaIBR* from *Pca* RM system	this study
pPF1439	*eyfp* reporter plasmid for insertion of *pcaIIR* promoter; IPTG-inducible *mCherry* reporter, pBR322 ori, Cm^R	(11)
pPF1525	pPF1117-derived, containing knockout cassette for entire *pcaI* RM system	this study
pPF1739	RK2 ori, *oriT*, Km^R; *mcherry* under T5-lac promoter control	Maureen Yin, unpublished
pPF1751	RK2 ori, *oriT*, Km^R; *zsgreen* under T5-lac promoter control	Maureen Yin, unpublished
pPF2860	pPF1439-derived reporter plasmid, *eyfp* combined with wild-type *pcaIIR* promoter	this study
pPF2861	pPF1439-derived reporter plasmid, *eyfp* combined with point-mutated *pcaIIR* promoter	this study
pPF2865	pBAD30-derived arabinose-inducible *pcaIM* expression plasmid	this study
pPF2870	pPF1117-derived, containing knockout cassette for *pcaIIR*	this study

Supplementary Table S4: Upregulated genes in *Pca*$^{\Delta RM}$.

**ORF** (F9W95_)	**predicted protein product or function**	**log$_2$-fold change**	**standard error**	p_{adj}
06460	HNH endonuclease	3.05	0.16	2.28E-76
13185	NADH oxidoreductase	1.34	0.21	5.86E-08
20145	LysE family translocator	1.25	0.25	9.48E-05
13190	hydroxylamine reductase Hcp	1.20	0.18	2.82E-08
16740	cytochrome c-type protein NrfB	1.15	0.21	9.88E-06
16730	cytochrome c nitrite reductase subunit NrfD	1.13	0.18	3.09E-07
16735	formate-dependent nitrite reductase complex subunit NrfC	1.06	0.17	1.53E-07
14060	4Fe-4S binding protein	1.01	0.18	1.16E-05
06380	aspartate ammonia-lyase AspA	0.92	0.27	3.34E-02
16725	heme lyase CcmF/NrfE family subunit	0.90	0.18	1.32E-04
16720	formate-dependent nitrite reductase complex subunit NrfF	0.89	0.24	1.18E-02
16745	nitrite reductase NrfA	0.86	0.16	2.51E-05
00475	AzlD domain-containing protein	0.83	0.25	3.37E-02
21245	metal-dependent phosphohydrolase	0.77	0.17	1.26E-03
21545	iron-sulfur cluster repair protein YtfE	0.75	0.20	1.17E-02
16715	formate-dependent nitrite reductase complex subunit NrfG	0.74	0.22	2.80E-02
01000	alkyl hydroperoxide reductase subunit F	0.71	0.21	3.63E-02
16500	MATE family efflux transporter	0.64	0.17	1.13E-02
08305	tellurite methyltransferase TehB	0.64	0.15	2.02E-03
03005	nucleotidyl transferase AbiEii/AbiGii toxin family protein	0.60	0.16	1.13E-02
18580	phosphate acetyltransferase	0.57	0.17	2.92E-02
19825	flavohemoprotein HmpA	0.56	0.15	1.05E-02
02800	HAMP domain-containing protein	0.53	0.15	1.70E-02
17870	D-serine/D-alanine/glycine transporter CycA	0.53	0.13	6.74E-03
08405	MHS family MFS transporter	0.52	0.14	1.05E-02
07705	DUF1176 domain-containing protein	0.50	0.14	2.27E-02
03030	ArsR family transcriptional regulator	0.50	0.11	1.92E-03
01775	siroheme synthase CobA	0.50	0.13	7.71E-03
21665	YggU family protein	0.49	0.15	4.93E-02
13250	ATP-binding/permease protein CydD	0.48	0.14	2.68E-02
10615	integrase/recombinase Int	0.47	0.14	3.94E-02
17660	motility protein B	0.46	0.11	5.82E-03
02245	5-amino-6-(5-phospho-D-ribitylamino)uracil phosphatase YigB	0.45	0.14	4.05E-02
19445	Vacuolating autotransporter toxin	0.44	0.14	4.34E-02
03865	tRNA uridine 5-carboxymethylaminomethyl modification enzyme MnmG	0.43	0.12	1.64E-02
19735	chaperone protein HscA	0.42	0.13	3.63E-02
17635	chemotaxis response regulator protein-glutamate methylesterase	0.41	0.12	2.80E-02
17180	rhodanese-related sulfurtransferase	0.40	0.12	4.44E-02
16430	pyridoxine/pyridoxamine 5′-phosphate oxidase PdxH	0.36	0.11	3.35E-02
01815	ribulose-phosphate 3-epimerase	0.34	0.10	4.88E-02

Supplementary Table S5: Downregulated genes in *Pca*$^{\Delta RM}$.

**ORF** (F9W95_)	**predicted protein product or function**	**log$_2$-fold change**	**standard error**	p_{adj}
06435	RtcB family protein	-1.58	0.47	3.63E-02
02970	ABC transporter permease subunit	-1.36	0.36	1.16E-02
02975	ABC transporter permease subunit	-1.34	0.34	8.12E-03
15645	Hrp pili protein HrpA	-1.17	0.24	1.93E-04
15875	threo-3-hydroxy-L-aspartate ammonia-lyase	-1.14	0.33	2.80E-02
04345	small heat shock protein IbpA	-1.12	0.22	9.48E-05
15700	type III secretion protein	-1.11	0.31	1.90E-02
10415	thioredoxin 1	-1.10	0.30	1.70E-02
08155	oxidoreductase	-1.04	0.28	1.58E-02
15685	EscD/YscD/HrpQ family type III secretion system inner membrane ring protein	-1.03	0.27	1.02E-02
15555	pectate lyase	-1.02	0.25	4.80E-03
15595	type III secretion protein HrpN	-1.02	0.27	1.16E-02
15545	DspFAvrF family protein	-0.98	0.25	7.56E-03
08200	insulinase family protein	-0.97	0.16	4.25E-07
00705	Na^+/H^+ antiporter NhaA	-0.97	0.24	7.67E-03
05435	host cell division inhibitor Icd-like protein	-0.92	0.27	2.80E-02
08195	TonB-dependent receptor	-0.91	0.15	3.40E-07
15560	DNA-binding protein	-0.89	0.26	2.86E-02
15690	EscN/YscN/HrcN family type III secretion system ATPase	-0.88	0.22	7.56E-03
15695	type III secretion protein	-0.88	0.25	2.80E-02
15675	YopN family type III secretion system gatekeeper subunit	-0.87	0.25	2.77E-02
10765	potassium-transporting ATPase ATP-binding subunit KdpB	-0.86	0.21	5.35E-03
01945	TonB-dependent siderophore receptor	-0.83	0.15	2.00E-05
07455	discoidin domain-containing protein	-0.76	0.20	1.18E-02
08205	ABC transporter ATP-binding protein/permease	-0.75	0.19	6.21E-03
01315	malate synthase A	-0.75	0.22	3.01E-02
08490	FhuE receptor	-0.73	0.19	1.18E-02
06750	isochorismatase	-0.72	0.18	5.80E-03
15550	AvrE-family type 3 secretion system effector	-0.71	0.21	3.54E-02
12960	TonB-dependent receptor	-0.71	0.09	4.09E-11
15610	EscC/YscC/HrcC family type III secretion system outer membrane ring protein	-0.69	0.20	3.09E-02
21730	PTS lactose/cellobiose transporter subunit IIA	-0.69	0.21	4.96E-02
16145	ShlB/FhaC/HecB family hemolysin secretion/activation protein	-0.68	0.20	2.71E-02
03615	Ail/Lom family outer membrane beta-barrel protein	-0.68	0.21	3.63E-02
15670	RNA polymerase sigma factor	-0.68	0.15	1.56E-03
05775	NAD(P)-binding protein	-0.67	0.15	8.78E-04
14625	aldehyde-alcohol dehydrogenase AdhE	-0.65	0.13	2.61E-04
06755	amino acid adenylation domain-containing protein	-0.61	0.13	3.51E-04
01940	Y4yA family PLP-dependent enzyme	-0.61	0.15	5.26E-03
10780	DUF2517 family protein	-0.60	0.18	3.05E-02
00700	transcriptional activator protein NhaR	-0.60	0.15	9.10E-03
17860	hypothetical protein	-0.58	0.18	4.02E-02
08080	exotoxin	-0.57	0.17	3.23E-02
06730	TonB-dependent receptor	-0.54	0.12	1.78E-03
06530	antitoxin YefM	-0.53	0.15	2.72E-02
01985	IclR family transcriptional regulator	-0.53	0.12	1.56E-03
11065	sugar phosphate isomerase/epimerase	-0.49	0.12	2.63E-03
10865	succinate dehydrogenase iron-sulfur subunit	-0.48	0.14	2.86E-02
00715	chaperone protein DnaK	-0.47	0.12	1.16E-02
14255	cupin domain-containing protein	-0.46	0.13	3.05E-02
17590	flagellar basal body rod protein FlgB	-0.46	0.14	4.21E-02
14845	arabinose ABC transporter substrate-binding protein	-0.46	0.11	7.61E-03
12395	TonB-dependent receptor	-0.45	0.12	1.13E-02
06295	type II toxin-antitoxin system RelE/ParE family toxin	-0.45	0.12	1.09E-02
10885	succinate-CoA ligase (ADP-forming) subunit alpha	-0.43	0.11	1.16E-02
06940	60 kDa chaperonin GroL	-0.43	0.12	2.20E-02
03050	transcriptional regulator	-0.43	0.11	1.05E-02
17600	negative regulator of flagellin synthesis FlgM	-0.39	0.11	1.70E-02
10875	dihydrolipoyllysine-residue succinyltransferase component of 2-oxoglutarate dehydrogenase complex OdhB	-0.38	0.10	1.09E-02
17405	RNA polymerase sigma factor FliA	-0.38	0.11	2.13E-02
17470	flagellar hook-basal body complex protein FliE	-0.37	0.09	6.74E-03
17420	GNAT family N-acetyltransferase	-0.37	0.11	2.89E-02
03815	D-ribose pyranase RbsD	-0.35	0.11	4.54E-02
17580	basal-body rod modification protein FlgD	-0.35	0.09	9.98E-03
13365	imidazole glycerol phosphate synthase subunit HisF	-0.35	0.10	3.30E-02
03800	ribose import binding protein RbsB	-0.30	0.09	3.26E-02
17570	flagellar basal body rod protein FlgF	-0.26	0.08	2.80E-02

SUPPLEMENTARY FIGURES

plasmid	origin	res	size (bp)	number of motifs		
				CCWGG	CCSGG	CCNGG
pPF953	ColE1	Tc	5,942	16	11	27
pPF781	p15A	Cm	3,868	13	14	27
pPF1122	ColE1	Cm	3,578	11	10	21
pPF1121	ColE1	Km	4,058	11	11	22
pQE80L-*oriT-mcherry*	ColE1	Ap	5,674	14	10	24
pTRB30	ColE1	Km	4,660	13	10	23

(vector used for transformation experiments in this study)

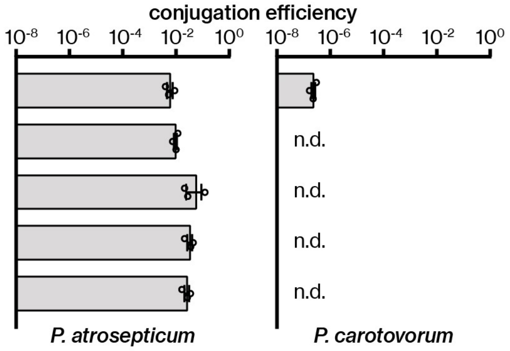

Supplementary Figure S1: Conjugation efficiency of different plasmids into *P. atrosepticum* SCRI1043 and *P. carotovorum* RC5297. Plasmid characteristics are indicated; res: antibiotic resistance marker (Tc: tetracycline; Cm: chloramphenicol; Km: kanamycin; Ap: ampicillin). Conjugation efficiencies are displayed as the mean and associated standard error, with independent replicates represented by dots; n.d.: not detected.

```
M.Pcal    96   FKFIDLFAGIGGVRLGFQNAGGACVFSSEYDKHAQQTYMRNHGEYP--------FGDITL  147
               F+FIDLFAGIGG+R GF++ GG CVF+SE++KHA +TY  NH   P          DITL
Eco Dcm   87   FRFIDLFAGIGGIRRGFESIGGQCVFTSEWNKHAVRTYKANHYCDPATHHFNEDIRDITL  146

M.Pcal    148  IDPD-------------YIPDHDILLAGFPCQPFSHAGL----KLG-----IDDTRGTLF  185
                  +             +IP+HD+LLAGFPCQPFS AG+     LG       DT+GTLF
Eco Dcm   147  SHKEGVSDEAAAEHIRQHIPEHDVLLAGFPCQPFSLAGVSKKNSLGRAHGFACDTQGTLF  206

M.Pcal    186  HDIANIIEKKKPKFALLENVKGLISHDKGFTLKVILKTLTRIGYSCNIPKDIIENGSTRK  245
                D+  II+ ++P   +LENVK L SHD+G T ++I++TL  +GY      D  +NG
Eco Dcm   207  FDVVRIIDARRPAMFVLENVKNLKSHDQGKTFRIIMQTLDELGYDV---ADAEDNGPD--  261

M.Pcal    246  IQELAKEMVLKSIDFGVPQNRQRIYIILWKDGELDKFEYP----SAC--DKSVCVGDILE  299
                       ++    F +PQ+R+RI ++ ++      K ++     S C   + V +  +L+
Eco Dcm   262  -----DPKIIDGKHF-LPQHRERIVLVGFRRDLNLKADFTLRDISECFPAQRVTLAQLLD  315

M.Pcal    300  RSPDPDLTISDRLWAGHQRRKVENKKNGKGFGFGLV--SSESSYTNTISARYYKDGSEIL  357
                  +    ++  LW    R   +++  G GFG+G+V  ++  S T T+SARYYKDG+EIL
Eco Dcm   316  PMVEAKYILTPVLWKYLYRYAKKHQARGNGFGYGMVYPNNPQSVTRTLSARYYKDGAEIL  375

M.Pcal    358  IDQGHD----------------NPRKISLREAARLQGF--PDD--FEPSKSKMQAYKQFG  397
               ID+G D                 PR+++ RE ARL GF  P +  F    S  QAY+QFG
Eco Dcm   376  IDRGWDMATGEKDFDDPLNQQHRPRRLTPRECARLMGFEAPGEAKFRIPVSDTQAYRQFG  435

M.Pcal    398  NS  399
               NS
Eco Dcm   436  NS  437
```

Supplementary Figure S2: M.PcaI and *E. coli* Dcm display similarity at the sequence level. BLAST alignment of the two proteins, with amino acid positions indicated at the beginning and end of each row and a consensus sequence given between the two protein sequences; + indicates biochemically similar amino acids.

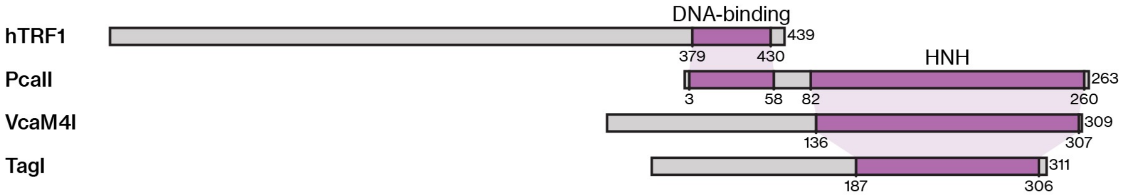

Supplementary Figure S3: PcaII contains a putative DNA-binding and an HNH domain. Domain alignments of PcaII with the DNA-binding domain of hTRF (12) and the HNH domains of VcaM4I (13) and TagI (14). Sequences aligned with high confidence using Phyre2 are shown in purple.

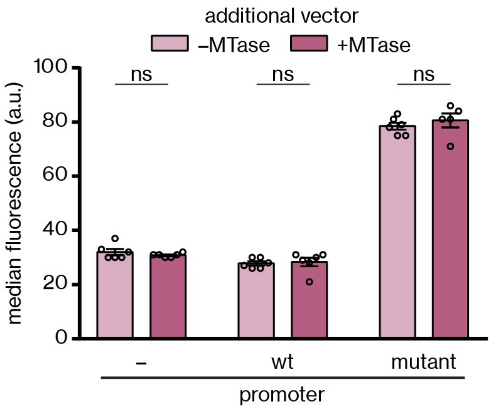

Supplementary Figure S4: In the *Pca*^{wt} background, overexpression of *pcaIM* has no effect on the *pcaIIR* promoter. Activity of the wild-type (wt) *pcaIIR* promoter (pPF2860) or a promoter variant with a 5′-CGTGG-3′ point mutation (pm) in the methylation motif (pPF2861), compared to an empty vector control (pPF1439), in the *Pca*^{wt} background in the presence of a second plasmid for expression of *pcaIM* or an empty vector (pPF2865 or pBAD30). Shown are the mean and associated standard error, with independent replicates represented by dots. Statistical significance was assessed using two-tailed unpaired *t*-tests; ns: $p>0.05$.

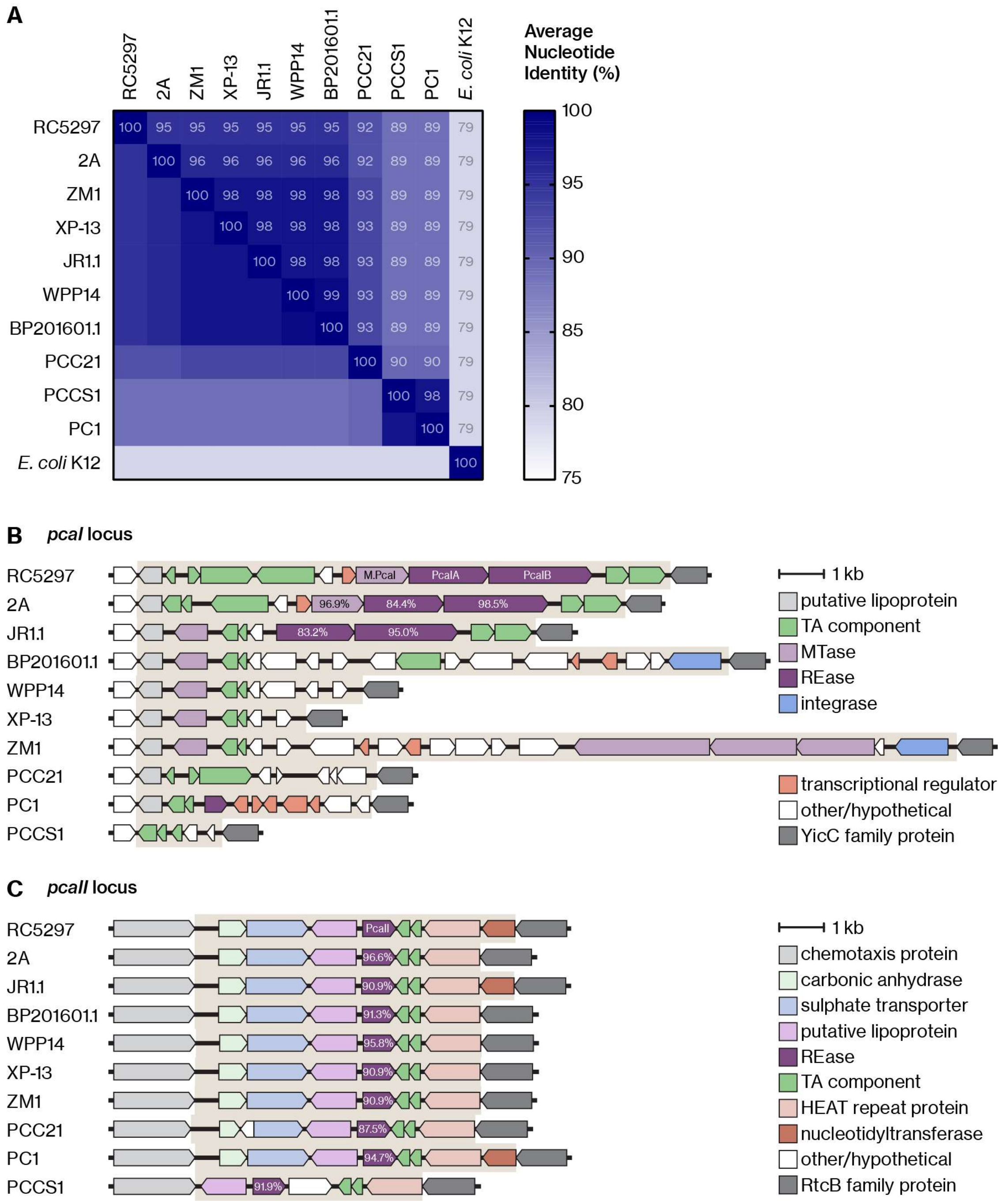

Supplementary Figure S5: *P. carotovorum* strains display different degrees of relatedness based on genome identity and have variable *pcaI* but conserved *pcaII* loci. A) Pairwise Average Nucleotide Identity (ANI) scores of various *P. carotovorum* strains and *E. coli* K12 as an outgroup. Depth of blue colour correlates with the degree of ANI, with numerical values indicating the percentage for each pair of strains. **B,C)** Overview of the loci corresponding to the RC5297 *pcaI* (**B**) and *pcaII* (**C**) loci across various *P. carotovorum* strains. The scale bar indicates a length of 1 kb and genes are coloured according to the legends beside. Genes encoding M.PcaI, PcaIA, PcaIB and PcaII are indicated for str. RC5297, with percentages in other strains indicating amino-acid identity to the RC5297 homologs. The light brown areas indicate the regions used for GC content determination shown in **Figure 7C**.

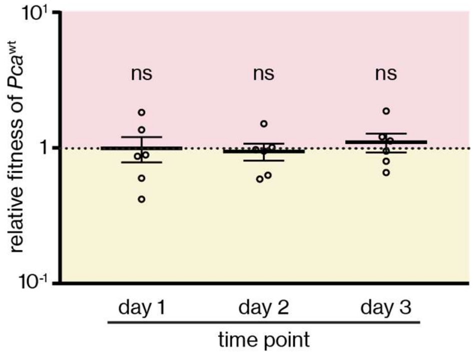

Supplementary Figure S6: Reciprocal use of the mCherry and ZsGreen fluorophores in a competition assay between *Pca*^{wt} and *Pca*$^{\Delta RM}$ also demonstrates no fitness difference. Relative fitness of *Pca*^{wt} (carrying the *mcherry* expression plasmid pPF1739) when competed against *Pca*$^{\Delta RM}$ (carrying the *zsgreen* expression plasmid pPF1751) for a period of three days. Shown are the mean and associated standard error, with independent replicates represented by dots. Statistical significance was assessed using a one-sample *t*-test against the hypothetical value 1, indicating no change in relative fitness; ns: p>0.05.

SUPPLEMENTARY METHODS

Construction of methyltransferase expression plasmids

To construct the plasmid pPF1375 for *pcaIM* expression, the MTase gene was first PCR-amplified from *Pca*^{wt} genomic DNA using the primer pair PF2630+PF2631. The PCR product was gel-purified and digested with MfeI and PstI, then purified and ligated with pTRB30 digested with the same enzymes. After transformation, colonies were PCR-screened using PF192+PF210 and the sequence of the plasmid was confirmed by DNA sequencing.

The *pcaIM* expression plasmid pPF2865 was constructed by first amplifying *pcaIM* from *Pca*^{wt} genomic DNA using PF5627+PF5628. The PCR product was digested with EcoRI and SalI and ligated with pBAD30 digested with the same enzymes. Transformant colonies were PCR-screened with PF138+PF139 and the correct sequence was confirmed by DNA sequencing.

Construction of *pcaIIR* promoter reporter plasmids

To construct the *pcaIIR* promoter reporter plasmids pPF2860 (wild-type promoter) and pPF2861 (point-mutated promoter), the promoter variants were first amplified from gBlocks PF5625 (wild type) and PF5626 (point mutation). Purified PCR products were digested with SpeI and BamHI and ligated with pPF1439 which had been cut with the same enzymes. Following transformation, colonies were PCR-screened using PF2517+PF2241 and plasmid sequences were confirmed by DNA sequencing.

Construction of *P. carotovorum* knockout plasmids and strains

Knockouts derived from *P. carotovorum* RC5297 (*Pca*^{wt}) were generated by allelic exchange mutagenesis. For the construction of strain PCF425 (*Pca*$^{\Delta R}$), which lacks the restriction endonuclease component of the RM system, upstream and downstream flanks were first PCR-amplified using primer pairs PF2698+PF2699 and PF2700+PF2701, respectively, with *Pca* genomic DNA as template. The two flanks were annealed and extended using PF2698+PF2701 and the resulting fragment was digested with the restriction enzymes NheI and NsiI. The digested fragment was then ligated into the suicide vector pPF1117, which requires a λ *pir*$^{+}$ host strain for replication. Therefore, these cloning steps were carried out using *E. coli* ST18 as the host for transformation. The resulting plasmid, pPF1424, was co-transformed with the *pcaIM* expression plasmid pPF1375 into *E. coli* ST18; MTase expression was required for efficient conjugation of pPF1424. Overnight cultures of the resulting strain were grown in the presence of the required antibiotics and supplements as well as IPTG for MTase induction. The cultures were used to conjugate pPF1424 into *Pca*^{wt}, with a donor:recipient ratio of 10:1. Colonies were restreaked twice on LBA + Cm. Next, overnight cultures were grown in the absence of Cm to allow plasmid loss, and culture dilutions were plated on LBA + 10% sucrose (w/v). The suicide vector contains *sacB*, a gene lethal to the

host if expressed in the presence of sucrose, hence this step selected against clones with the plasmid integrated in their genomes. Resulting colonies were restreaked on LBA + Cm to confirm loss of antibiotic resistance and PCR-screened using primers PF2857+PF2858. PCR products were sequenced to confirm the knockout.

The procedure for the construction of strain PCF433 ($Pca^{\Delta RM}$) followed the same protocol with minor modifications. In this case, the upstream flank for the homology repair template was generated using primers PF2916+PF2917 (with the downstream flank as above). The flanks were annealed and extended using PF2916+PF2701 and cloned into pPF1117 to yield pPF1525, which was transformed into *E. coli* ST18 and conjugated into PCF425. Note that presence of the *pcaIM* expression plasmid was not required in this case due to the absence of the REases in the recipient. With the remaining steps as above, the final knockout was confirmed using primers PF2918+PF2858.

For the construction of PCF890 and PCF891 – *pcaIIR* knockouts in the Pca^{wt} and $Pca^{\Delta RM}$ backgrounds, respectively – the upstream flank was generated using PF5689+PF5606 and the downstream flank using PF5607+PF5690. Cloning the flanks into pPF1117 yielded pPF2870, which was subsequently conjugated from *E. coli* ST18 into Pca^{wt} and $Pca^{\Delta RM}$; in the case of Pca^{wt}, this again required the presence of the *pcaIM* expression plasmid pPF1375. The remaining steps were performed as described above and the final knockouts were confirmed using PF5616+PF5691.

REFERENCES

1. Tovkach, F.I. (2002) Study of *Erwinia carotovora* Phage Resistance with the Use of Temperate Bacteriophage ZF40. *Microbiology*, **71**, 72-77.
2. Bell, K.S., Sebaihia, M., Pritchard, L., Holden, M.T.G., Hyman, L.J., Holeva, M.C., Thomson, N.R., Bentley, S.D., Churcher, L.J.C., Mungall, K. *et al.* (2004) Genome sequence of the enterobacterial phytopathogen *Erwinia carotovora* subsp. *atroseptica* and characterization of virulence factors. *Proc Natl Acad Sci USA*, **101**, 11105-11110.
3. Taylor, R.G., Walker, D.C. and McInnes, R.R. (1993) *E.coli* host strains significantly affect the quality of small scale plasmid DNA preparations used for sequencing. *Nucleic Acids Res*, **21**, 1677-1678.
4. Thoma, S. and Schobert, M. (2009) An improved *Escherichia coli* donor strain for diparental mating. *FEMS Microbiol Lett*, **294**, 127-132.
5. Jackson, S.A., Fellows, B.J. and Fineran, P.C. (2020) Complete Genome Sequences of the *Escherichia coli* Donor Strains ST18 and MFD*pir*. *Microbiol Resour Announc*, **9**, e01014-01020.
6. Guzman, L.-M., Belin, D., Carson, M.J. and Beckwith, J. (1995) Tight Regulation, Modulation, and High-Level Expression by Vectors Containing the Arabinose P_{BAD} Promoter. *J Bacteriol*, **177**, 4121-4130.
7. Richter, C., Dy, R.L., McKenzie, R.E., Watson, B.N.J., Taylor, C., Chang, J.T., McNeil, M.B., Staals, R.H.J. and Fineran, P.C. (2014) Priming in the Type I-F CRISPR-Cas system triggers strand-independent spacer acquisition, bi-directionally from the primed protospacer. *Nucleic Acids Res*, **42**, 8516-8526.
8. Przybilski, R., Richter, C., Gristwood, T., Clulow, J.S., Vercoe, R.B. and Fineran, P.C. (2011) Csy4 is responsible for CRISPR RNA processing in *Pectobacterium atrosepticum*. *RNA Biol*, **8**, 517-528.
9. Patterson, A.G., Jackson, S.A., Taylor, C., Evans, G.B., Salmond, G.P.C., Przybilski, R., Staals, R.H.J. and Fineran, P.C. (2016) Quorum Sensing Controls Adaptive Immunity through the Regulation of Multiple CRISPR-Cas Systems. *Mol Cell*, **64**, 1102-1108.
10. Jackson, S.A., Birkholz, N., Malone, L.M. and Fineran, P.C. (2019) Imprecise Spacer Acquisition Generates CRISPR-Cas Immune Diversity through Primed Adaptation. *Cell Host Microbe*, **25**, 250-260.
11. Smith, L.M., Jackson, S.A., Malone, L.M., Ussher, J.E., Gardner, P.P. and Fineran, P.C. (2021) The Rcs stress response inversely controls surface and CRISPR–Cas adaptive immunity to discriminate plasmids and phages. *Nat Microbiol*, **6**, 162-172.
12. Court, R., Chapman, L., Fairall, L. and Rhodes, D. (2005) How the human telomeric proteins TRF1 and TRF2 recognize telomeric DNA: a view from high-resolution crystal structures. *EMBO Rep*, **6**, 39-45.
13. Pastor, M., Czapinska, H., Helbrecht, I., Krakowska, K., Lutz, T., Xu, S.-y. and Bochtler, M. (2021) Crystal structures of the EVE-HNH endonuclease VcaM4I in the presence and absence of DNA. *Nucleic Acids Res*, **49**, 1708-1723.
14. Kisiala, M., Copelas, A., Czapinska, H., Xu, S.-Y. and Bochtler, M. (2018) Crystal structure of the modification-dependent SRA-HNH endonuclease TagI. *Nucleic Acids Res*, **46**, 10489-10503.
